# Climate change could overturn bird migration: Transarctic flights and high-latitude residency in a sea ice free Arctic

**DOI:** 10.1038/s41598-019-54228-5

**Published:** 2019-11-28

**Authors:** Manon Clairbaux, Jérôme Fort, Paul Mathewson, Warren Porter, Hallvard Strøm, David Grémillet

**Affiliations:** 1 CEFE UMR 5175, CNRS – Université de Montpellier – Université Paul-Valéry Montpellier – EPHE, Montpellier, France; 2 Littoral Environnement et Sociétés (LIENSs), UMR 7266 CNRS-Université de La Rochelle, La Rochelle, France; 3 Department of Integrative Biology, University of Wisconsin, Madison, WI USA; 4 Norwegian Polar Institute, Fram Centre, P.O. Box 6606, Langnes, 9296 Tromsø Norway; 5 Percy FitzPatrick Institute, DST/NRF Centre of Excellence, University of Cape Town, Rondebosch, South Africa

**Keywords:** Animal migration, Biogeography

## Abstract

Climate models predict that by 2050 the Arctic Ocean will be sea ice free each summer. Removing this barrier between the Atlantic and the Pacific will modify a wide range of ecological processes, including bird migration. Using published information, we identified 29 arctic-breeding seabird species, which currently migrate in the North Atlantic and could shift to a transarctic migration towards the North Pacific. We also identified 24 arctic-breeding seabird species which may shift from a migratory strategy to high-arctic year-round residency. To illustrate the biogeographical consequences of such drastic migratory shifts, we performed an in-depth study of little auks (*Alle alle*), the most numerous artic seabird. Coupling species distribution models and climatic models, we assessed the adequacy of future wintering and breeding areas for transarctic migrants and high-arctic year-round residents. Further, we used a mechanistic bioenergetics model (Niche Mapper), to compare the energetic costs of current little auk migration in the North Atlantic with potential transarctic and high-arctic residency strategies. Surprisingly, our results indicate that transarctic little auk migration, from the North Atlantic towards the North Pacific, may only be half as costly, energetically, than high-arctic residency or migration to the North Atlantic. Our study illustrates how global warming may radically modify the biogeography of migratory species, and provides a general methodological framework linking migratory energetics and spatial ecology.

## Introduction

The Arctic environment is highly seasonal and bird breeding phenologies closely match enhanced spring and summer resource availability^1^. Most species subsequently leave the Arctic to winter at lower latitudes, resulting in the migration of billions of individuals. Migration and overwintering are periods during which high mortality occurs^2,3^. Long-distance flights and winter habitat quality may also have carry-over effects on subsequent breeding success^4,5^. Overall, migration greatly contributes to shaping bird population dynamics^3,6^. Studying arctic bird migration at the individual, population, species and community levels is therefore a major research objective, which has greatly benefited from recent developments in migration tracking technologies. These technologies allow a better understanding of how birds might choose migratory routes and wintering areas, and help analyze the interplay between genetic and phenotypic plasticity in shaping bird responses to geographical and ecological barriers^7^, intra- and interspecific competition^8^, as well as the consequences of environmental change^9^.

Climate change has direct and indirect effects on birds^10^ and migratory species are particularly sensitive. Notably, altered climatic conditions can modify migratory phenologies^11,12^ and result in shifting wintering and/or breeding areas, with consequences for migratory distances^3,13^. Global changes may even result in species/populations switching from a migratory to a resident strategy, and *vice versa*^3,14^.

Global warming is fastest in the Arctic, with a temperature increase more than twice the world’s average^15^. This has marked impacts on the arctic cryosphere: The central part of the Arctic Basin, where some areas have been permanently covered by multi-year sea ice for at least the last 5,500 years^16^, is supposed to become completely sea ice free each summer before the mid-21^st^-century^15,17^. Such a drastic habitat modification will have major consequences for large scale ocean circulation^18,19^ but also for Arctic Ocean acidity^20^ and productivity^21,22^, with impacts on ecological processes^23–25^.

Former glacial cycles governed transarctic exchanges between Pacific and Atlantic biota across time, leading to population mixing or isolation, and shaping evolution^19^. Thereby, colonization from the Pacific into the high Arctic and the North Atlantic already occurred in the mid/end Pliocene, induced by mild arctic conditions and ended by sea ice expansion^26^.

Currently, Arctic sea ice is an ecological barrier for migratory birds. Henningsson and Alerstam^27^ also rated transarctic migration as particularly difficult for birds because of navigational issues (but see^28,29^) and of the lack of stop-over sites. With sea ice constraining the availability of stop-over sites, more costly and risky non-stop transarctic flights are therefore unlikely. Conversely, migration along sea ice edges at the periphery of the Arctic Basin seems much more widespread^28^. Also, radar studies and direct observations demonstrated that several species of seabirds are capable of crossing the Arctic Basin^30,31^ as already observed in fishes^32^ and marine mammals^31^.

Re-creating sea ice free conditions favorable for transarctic exchanges^26^, climate change is in the process of drastically modifying constraints set upon arctic bird migration by sea ice. Indeed, Vermeij and Roopnarine^26^ predicted that a sea ice free Arctic Basin in summer will lead to enhanced transarctic migrations between the Atlantic and the Pacific oceans. Concomitantly with shifting migratory routes and wintering areas, some arctic-breeding bird species may also become year-round residents. High latitudes and the associated polar night has long been thought to impose a major constraint upon such a strategy, yet a series of recent studies demonstrated that birds may cope surprisingly well with very low light levels^33–35^.

In this context, the objectives of this study were to: (1) Determine which birds species could switch to transarctic bird migration and/or arctic year-round residency as a result of a decreasing sea ice cover within the Arctic Basin. (2) Assess the adequacy of future wintering and breeding habitats in the context of these two new migratory strategies. (3) Compare the energetics of current bird migration in the North Atlantic, with those linked to potential transarctic and high-arctic residency strategies.

As our aim was to study the impact of reduced sea ice cover on the propensity of birds to become transarctic migrants and/or year round residents, we focused on coastal and marine species. We thereby assumed that they are more directly impacted by a vanishing arctic sea ice cover. With respect to transarctic migration, we narrowed the range of studied species by selecting those which are pelagic during winter. Indeed, those species will benefit the most from a sea ice free Arctic Basin in future summers, and we assumed that they would consequently be the most prone to engage in new transarctic migrations. We assumed that for terrestrial or coastal birds with land based feeding habits, the sea would represent the same ecological barrier as an un-melted Arctic Basin.

Even if species are ecophysiologically capable of engaging in new migratory strategies, shifting to residency or to new transarctic migration induces the use of new breeding and/or wintering areas. Modeling of those future habitats is needed to assess their adequacy with potential new migratory strategies, for each species concerned. To this aim, we propose a methodological framework based on a mechanistic bioenergetics modelling (Niche Mapper), which we applied to little auks (*Alle alle*) as an example.

This species was chosen because the little auk is the most numerous seabird in the North Atlantic Arctic (population estimated at 40–80 million individuals^36^), with significant impact on terrestrial and marine trophic networks^37^ and an acknowledged sensitivity to environmental changes^38–41^. On the basis of its morphological and ecophysiological traits, we short-listed the little auk as a likely candidate for future year-round residency in the high Arctic, and/or for new transarctic migration (see Methods), from the North Atlantic into the Pacific.

## Methods

### Species selections

We defined the Arctic according to boundaries set by the Arctic Council and its working groups, notably the Conservation of Arctic Fauna and Flora (CAFF; https://www.caff.is)^42^. Following CAFF^42^, we selected coastal and marine birds among 316 migratory/partially migratory bird species whose breeding ranges overlapped by at least 5% with the arctic region. We assumed that bird species which are currently year-round residents of the Arctic would remain so. Indeed, shifting from a residency to a migratory strategy is far less frequent than the opposite shift^3^. Finally, even though poleward shifts in bird distributions do occur in response to climate change^43,44^ we did not include new species that may migrate into the Arctic in summer as a consequence of global warming. This would go beyond the scope of our current analysis, but would certainly be a valid target for future work.

#### Selection of potential new resident arctic bird species

We narrowed the range of studied species, by selecting those which primarily use coastal and marine habitats. Even in a climate change context high latitude photoperiods will remain unchanged and arctic winter residents will always have to cope with the polar night. We therefore further reduced our sample to species, or family of species, for which nocturnal activities (in particular foraging) have been described in literature, indicating that the considered species are potentially anatomically and ecophysiologically capable of surviving the polar night.

#### Selection of bird species susceptible to shift to a transarctic migration

We used the CAFF list of migrant arctic breeding birds (see above), and selected species with a primarily pelagic habitat during winter.

#### Predicting little auk’s future habitats with ecological niche modeling

**Figure 1:**
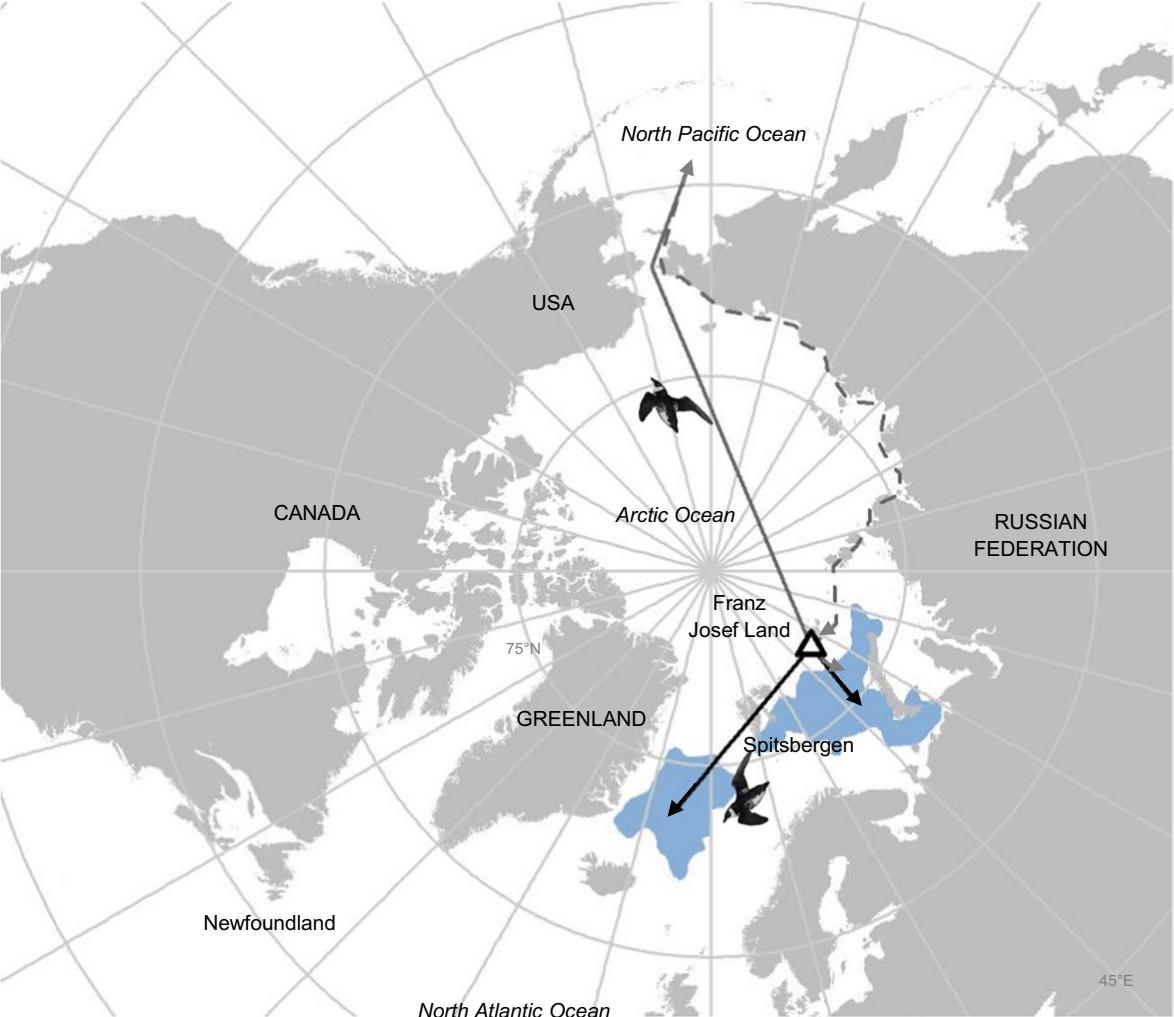
Current (black arrows) and future (grey arrows) migratory strategies of little auks breeding in Franz Josef Land (white triangle). In March, the return journey from Pacific could be made directly (grey arrows) or by by a peripheral flyway (grey dashed arrows). Their current known wintering areas (http://www.seapop.no/en/seatrack/) are in blue. Graticules are set at a 15° interval and the map is projected as North Pole Lambert Azimuthal Equal Area. Little auks drawings used in this figures were extracted from Richard Crossley’s picture (available online under CC-BY-SA license https://creativecommons.org/licenses/by-sa/2.0/legalcode at https://commons.wikimedia.org/wiki/File:Little_Auk_from_the_Crossley_ID_Guide_Britain_and_Ireland.jpg). This map has been made using R software (version 3.5.1, https://cran.r-project.org/) thanks “maptools”,“rgdal”,“rgeos” and “sp” packages.

Little auks mainly breed (May to August) in Greenland, Svalbard and the Russian Western Arctic, and currently migrate southwards into the Atlantic, with at-sea wintering areas (October to February) ranging from the Barents Sea to Newfoundland^45^ (See Supplemental Materials I). Following the aforementioned species selection, we short-listed the little auk as a likely candidate for future year-round residency in the high Arctic, and/or for new transarctic migration, from the North Atlantic into the Pacific (Fig. 1).

Current and future little auk nesting, summer foraging and wintering habitat distributions were modelled with ‘biomod2’^46^, which draws from current occurrences to predict suitable habitats in space and time, on the basis of environmental conditions.

#### Little auk occurrence data

Current occurrence data were direct observations drawn from three open access databases, the Global Biodiversity Information Facility (https://www.gbif.org/), the Ocean Biogeographic Information System-Spatial Ecological Analysis of Megavertebrate Population (http://seamap.env.duke.edu/) and the North Pacific Pelagic Seabird Database (https://www.usgs.gov/centers/asc/science/north-pacific-pelagic-seabird-database?qt-science_center_objects=0#qt-science_center_objects), and complemented with information on breeding locations from the literature^47–49^ and from the Norwegian Polar Institute^50^ (Russian data excluded). Only dated and located data for which environmental variables were available (see below), were conserved and duplicates deleted. Museum data weren’t considered.

Overall, we used respectively 67, 68 and 580 occurrences to model nesting, summer foraging and wintering habitats.

#### Environmental data

To predict nest site distributions, we used monthly mean air surface temperatures from 1948 to 2018 retrieved from National Oceanic and Atmospheric Administration (NOAA)’s 0.5° Global Historical Climatology Network version 2/Climate Anomaly Monitoring System. Since little auks breed underground and are limited by snow cover in their access to nest cavities, we calculated the percentage of time during which the ground was covered by snow two months before, and during the breeding period using the National Snow and Ice Data Center (NSIDC)’s IMS 4 km Daily Northern Hemisphere Snow and Ice Analysis between 2006 and 2017. Also, since little auks are central-place foragers during the breeding season, and have a constrained foraging range during that period, we created a discrete variable to deal with the distance from the coast (<10 km, <20 km, <50 km, <100 km, <200 km or > =200 km).

Previous work showed that wintering little auks are significantly constrained by air temperatures^48^. For marine areas, we therefore used monthly mean air surface temperature data from 1960 to 2017 retrieved from NOAA 1° International Comprehensive Ocean-Atmosphere Data Set (ICOADS). Since bathymetry and sea ice constrain little auk foraging^51,52^, we calculated the slope of the bathymetry using General Bathymetric Chart of the Oceans (GEBCO) 30 arc-second interval grid and used monthly sea ice concentration data (1978–2017) from the 25 km*25 km NOAA/NSIDC Climate Data Record of Passive Microwave Sea Ice Concentration. Used variables were not correlated as tested with a 0.8 threshold in a Pearson pairwise correlation test^53^ and a threshold of 10 for the variance inflation factor analysis. All environmental data were interpolated linearly on a 0.1° spatial grid, in the Northern Hemisphere. Environmental values were extracted for the year and month corresponding to each occurrence data.

#### Climate models

To make future predictions, environmental variables (see above) were considered under Intergovernmental Panel on Climate Change (IPCC)’s RCP8.5 scenario using four climatic models (HadGEM2-CC, HadGEM2-ES, ACCESS1.0 and ACCESS1.3) considered as performant (reasonably simulating recent past climate) when predicting future Arctic climates, in particular the cryosphere^17^.

#### Modeling little auk distributions

We used a model averaging approach in the ‘biomod2’ package in R^46^ with Boosted Regression Tree (BRT), Random Forest (RF), Classification Tree Analysis (CTA) and Flexible Discriminant Analysis (FDA) algorithms which deal in the same way with pseudo-absences parametrization^54^. Beyond existing presence data, we generated five sets of 1000 random pseudo-absences outside of current range (SRE method) and ten sets of 100 random pseudo-absences outside of a 2° area around each presence data, for wintering and for summer nesting/foraging distribution modeling, respectively. Pseudo-absences were time-stamped using the same temporal distribution as occurrence data, and environmental variables were extracted according to each location and date. We performed three runs for each set of pseudo-absences, with each distribution modeling and each ‘biomod2’ algorithm. For each run, outputs were assessed with the True Skill Statistic (TSS) and the importance of each environmental variables was calculated. Finally, for each distribution model, all obtained models (number of algorithms *3*number of pseudo-absence data sets) were weighted with TSS, and averaged to yield a single ensemble model. Those final models were evaluated with the continuous Boyce index^55^, which assess presence-only predictions and vary between -1 and 1, with 1 indicating good to perfect predictions^56^. Each final distribution model was then projected across space and time to map little auk potential distribution. For each future distribution projection, we calculated the coefficient of variation between climatic models.

We considered an area suitable for little auks when its probability of suitability (habitat suitability index) was higher than 0.9 (a high conservative threshold set with ‘biomod2’^46^). For distributions related to each climatic model, we assumed that suitable nesting sites within 200 km of suitable foraging areas were potential breeding areas. Indeed, the maximum foraging range for little auks during the breeding period rarely exceed 200 km^38,52,57^. Because environmental variables available in ACCESS 1.0 and ACCESS1.3 climatic models did not allow nesting distribution predictions, we used nesting sites obtained with the HadGEM2-CC and HadGEM2-ES climatic models. Further, we assumed that resident wintering little auks would remain in marine areas within 250 km of potential or known breeding sites.

### Energetic consequences of future migratory strategies

To calculate present and future little auk energy requirements according to each migratory strategy (current migration, residency or transarctic migration), we used Niche Mapper (see^58–60^).This mechanistic model contains a microclimate module, which provides environmental data for the immediate surroundings of the animal, and an animal module, which integrates outputs from the microclimate model with animal morphological, behavioral and physiological characteristics. Those modules are used to solve heat balance equations between the animal’s body and its surroundings, and estimate the metabolic rate required for the animal to remain in a thermal steady-state. Niche Mapper simulations were performed for the little auks population breeding in Franz Josef Land (Russian Federation), because both winter residency and transarctic migration are plausible for birds from this locality (see Results below) and their current wintering areas are known (Fig. 1). Niche Mapper has been previously used to model little auk wintering energetics^61,62^, and we built upon this prior work, notably using a majority of the same input values for bird morphological and physiological characteristics.

We modelled current and future little auk energy requirements during their migratory journey (in September and March) and wintering phase (October to February) according to three scenarios: (1) Current migration: At their current wintering areas in the North Atlantic (defined as the centroid of kernel distribution available on the SEATRACK website, see also Fig. 1). (2) Transarctic migration: At potential future wintering locations in the North Pacific, corresponding to the closer area predicted as suitable for the four climatic models using ‘biomod2’ (see previous section and results) (Fig. 1). (3) Residency: Within 250 km of their potential future breeding site in Franz Josef Land, in areas predicted as suitable using ‘biomod2’ (Fig. 1). For each strategy, the migratory flyway used in September was considered as the straight line between the colony and the wintering location, avoiding flights >100 consecutive km across land (Fig. 1). In the spring, the Arctic Basin is predicted to remain iced until much later in the 21^st^ century, and it is unclear whether little auks would engage in a direct flight to the Atlantic, or will perform a loop migration, whereby the return journey will use polynyas peripheral to the Arctic Basin as stop-over sites. Both case were studied, by considering a direct flyway (the same as in September) or a peripheral one, the latter corresponding to a path minimizing the time spend flying above areas dense in sea-ice (Fig. 1). For the current and residency strategies, the spring return journey is supposed to be the same as in September.

All required current and future environmental variables were retrieved from climatic models previously described. Outputs from climatic models were averaged on a 0.1° spatial grid for each environmental variable across 2006–2017 for the current scenario and across 2050–2059 for future predictions. Environmental values between October and February were then extracted for each strategy at the wintering location. Environmental conditions experienced during the migratory journey (in September and March) were calculated as the average of environmental values encountered during the trip for those months. Percentage of time spent flying per day during this travel was calculated assuming that birds migrated in one month, with an average flight speed of 13 m.s^−1^ ^63^. For each scenario, energetic costs obtained were averaged for the four climatic models and standard deviations between them were calculated.

All input data are available in Supplemental Materials II.

## Results

### Species selections

Among the 449 species which breed or have bred in the Arctic^42^, 359 (80%) have a breeding range which overlaps to >5% with the Arctic as defined by CAFF^42^. Among those, 316 (88%) are migrants or partial migrants (see Supplemental Materials III), and belong to 44 families (see Supplemental Materials III). During winter, 29 of those species are pelagic (essentially alcids, gulls and skuas) and another 37 (mainly ducks and gulls) utilize costal marine habitats (see Supplemental Materials III). Only 24 (see Supplemental Materials IV) of those 66 species are likely to remain active during the polar night, and may become year-round residents to the Arctic in the future. Alcids and gulls represent 42% of those species but some ducks, cormorants, petrels, shearwaters and loons, are potential future residents. Overall, our bibliography study indicated that only 29 pelagic species (6.5% of all arctic breeding species) are potential candidates for future transarctic migrations (see Supplemental Materials III).

### Predicting current and future little auk habitats

**Figure 2:**
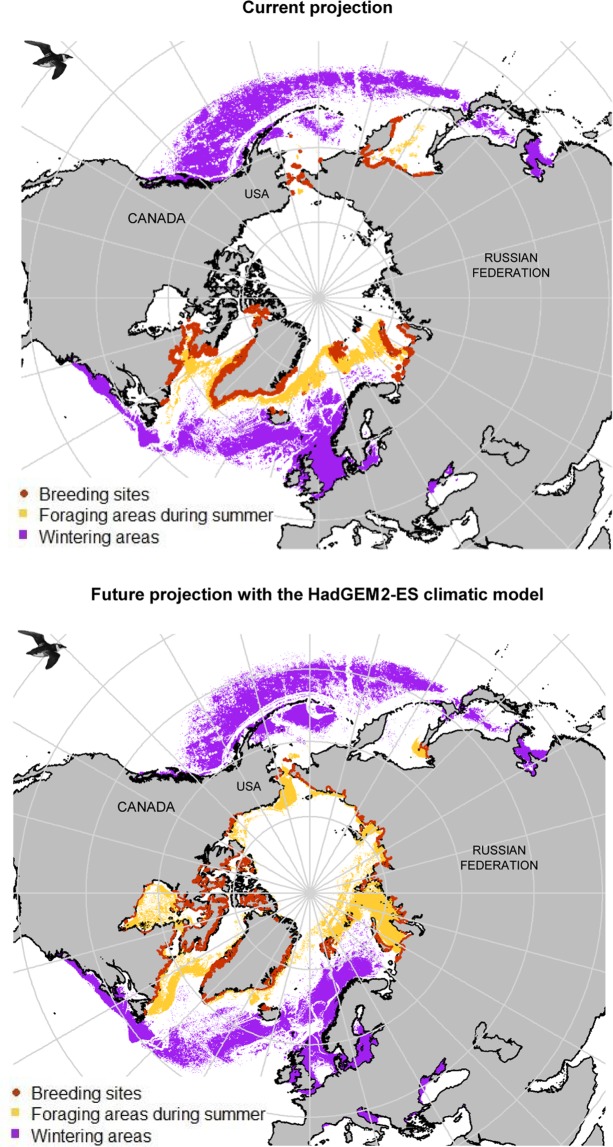
Potential suitable (suitability likelihood > 0.9) little auk habitats for present (2000–2017) and future (2050–2059, HadGEM2-ES climatic model, RCP 8.5 scenario) projections. This map has been made using R software (version 3.5.1, https://cran.r-project.org/) thanks “maptools”,“rgdal”,“rgeos” and “sp” packages. Little auks drawings used in this figures were extracted from Richard Crossley’s picture (available online under CC-BY-SA license https://creativecommons.org/licenses/by-sa/2.0/legalcode at https://commons.wikimedia.org/wiki/File:Little_Auk_from_the_Crossley_ID_Guide_Britain_and_Ireland.jpg).

**Figure 3:**
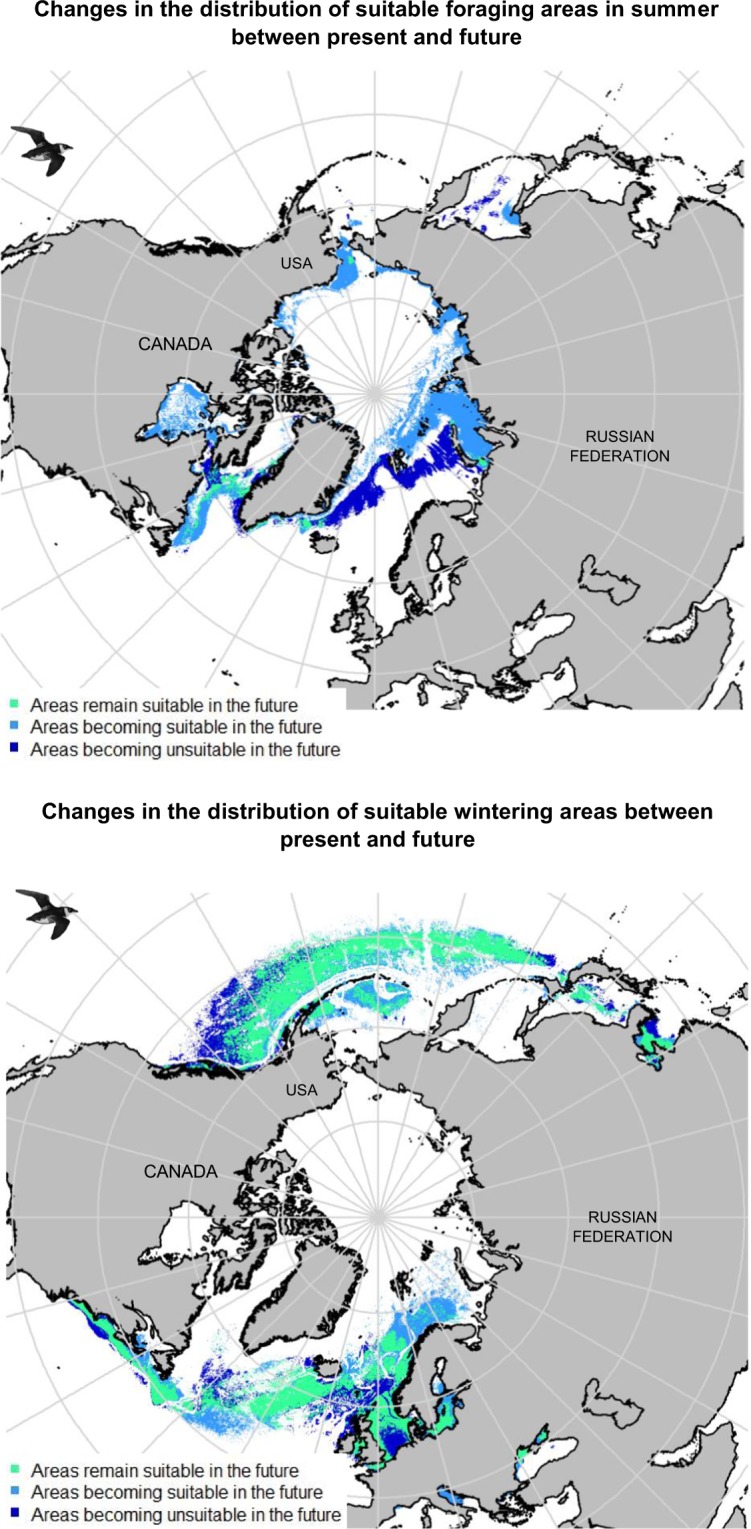
Changes in the potential distribution of suitable foraging and wintering habitats between present (2000–2017) and future (2050–2059, HadGEM2-ES climatic model, RCP 8.5 scenario) projections. This map has been made using R software (version 3.5.1, https://cran.r-project.org/) thanks “maptools”,“rgdal”,“rgeos” and “sp” packages. Little auks drawings used in this figures were extracted from Richard Crossley’s picture (available online under CC-BY-SA license https://creativecommons.org/licenses/by-sa/2.0/legalcode at https://commons.wikimedia.org/wiki/File:Little_Auk_from_the_Crossley_ID_Guide_Britain_and_Ireland.jpg).

**Figure 4:**
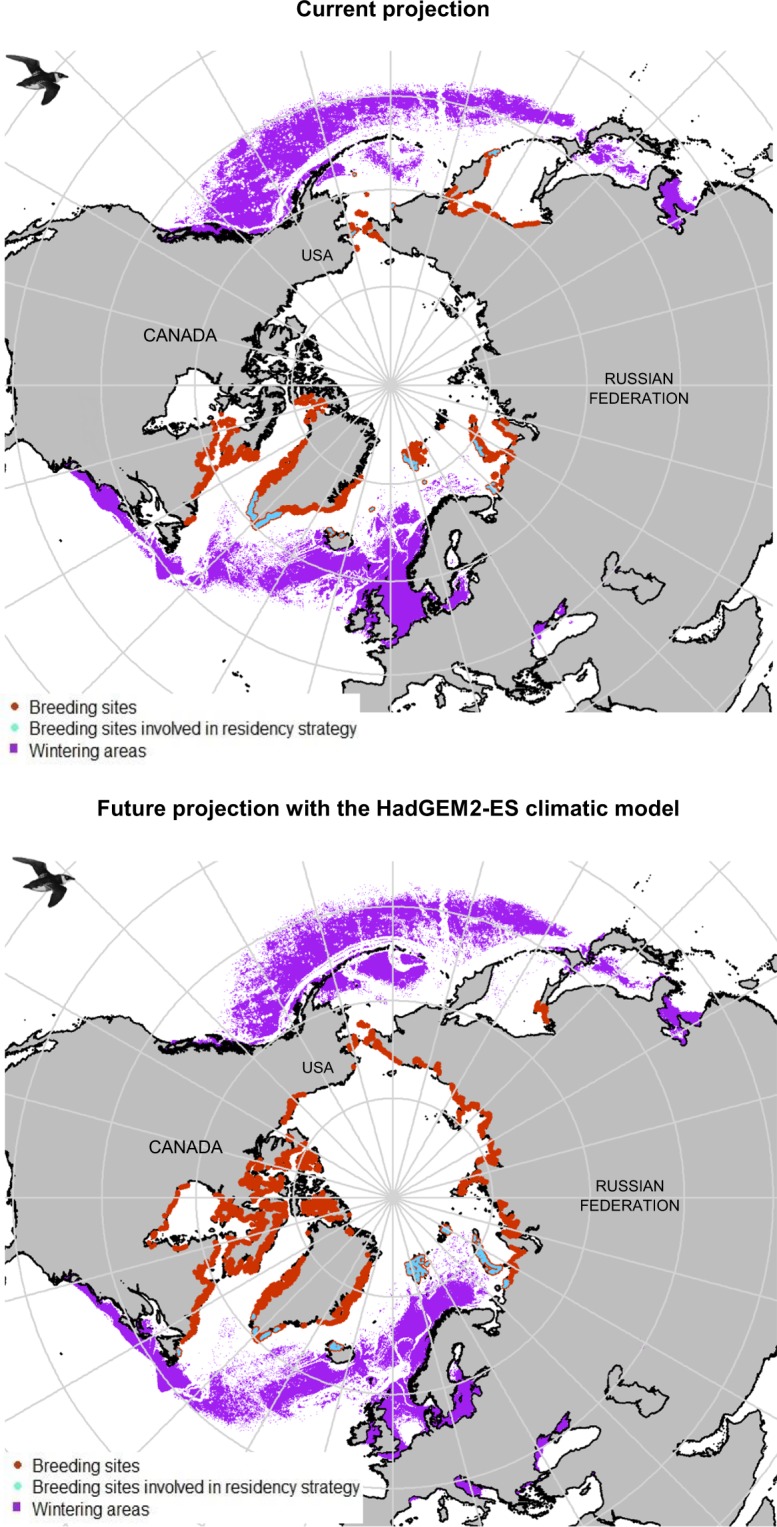
Potential suitable (suitability likelihood > 0.9) little auk breeding habitats involved in the residency strategy, currently (2000–2017) and in the future (2050–2059, HadGEM2-ES climatic model, RCP 8.5 scenario). This map has been made using R software (version 3.5.1, https://cran.r-project.org/) thanks “maptools”,“rgdal”,“rgeos” and “sp” packages. Little auks drawings used in this figures were extracted from Richard Crossley’s picture (available online under CC-BY-SA license https://creativecommons.org/licenses/by-sa/2.0/legalcode at https://commons.wikimedia.org/wiki/File:Little_Auk_from_the_Crossley_ID_Guide_Britain_and_Ireland.jpg).

Modelled current little auk habitats are presented in Fig. 2 and Supplemental Materials V. All averaging models concerning the breeding and wintering periods had a continuous Boyce Index close to 1 (0.823 and 0.769 for nesting and foraging areas respectively and 0.936 for winter area). According to ‘biomod2’, air temperature was the main driver of little auk marine distributions, whereas distance from the coast was the main driver of nesting distributions during the breeding season. During winter, highest suitability likelihoods were recorded both in the North Atlantic and in the North Pacific with some potential wintering hotspots in the North Sea and the Labrador Sea, which are in agreement with observed occurrences. During summer, predicted foraging areas seem to follow the sea ice edge, especially off East Greenland. Most known colonies were adequately predicted by model outputs, but the model seems to overfit in eastern Canada, by predicting suitable little auk habitat in regions where little auks do not breed. Future little auk habitats assessed according to the four climatic models are presented in Fig. 2 and Supplemental Materials V. The coefficient of variation map (Supplemental Materials VI) comparing model outputs shows their general concurrence. Overall, climate change is predicted to cause loss or gain of suitable little auk habitats, depending on the region (see Fig. 3 and Supplemental Materials V): For example, the Pacific Ocean off British Columbia (Canada) will become unsuitable for overwintering little auks, whereas the Barents Sea will become increasingly suitable. During summer, suitable foraging areas are predicted to shift northward, both in the Atlantic and in the Pacific. On land, breeding distributions are also predicted to shift northwards in response to climate change. Crucially, the main breeding area of Thule in Northwest Greenland, which currently hosts half of the the little auk world population, is predicted to become unsuitable according the climatic model HadGEM2-CC. Finally, model outputs suggested that shifting to transarctic migration towards the Pacific is a potential option for North Atlantic little auks. However, predicted migratory distance varied considerably, depending on the climatic model considered. Year-round high-arctic residency is also predicted to occur in the future, close to some nesting sites (Supplemental Materials V and Fig. 4).

### Energy requirements linked to future migratory strategies

**Figure 5:**
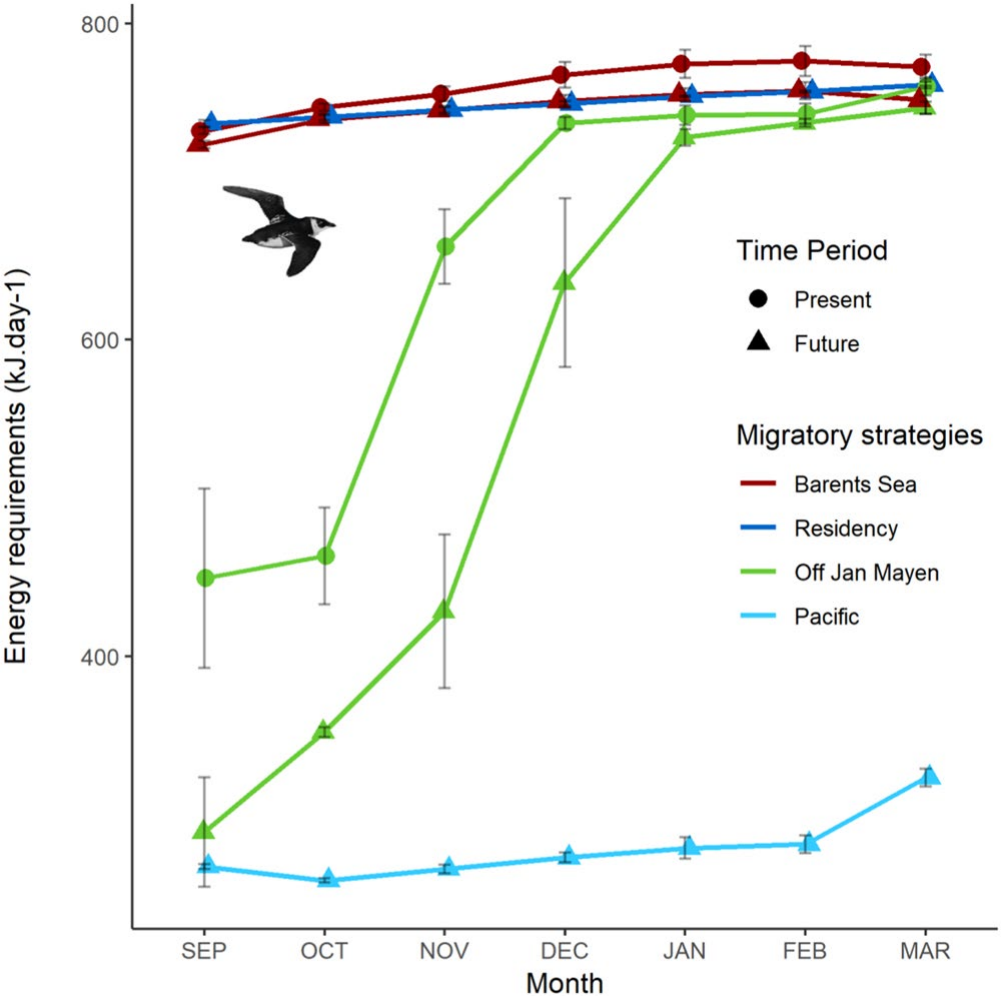
Average daily energy requirements (in kJ.day^−1^) for each month along the winter period according to different migratory strategies (see Fig. 1). ‘Barents Sea’ is for birds wintering in the Barents Sea, just South of their breeding areas, ‘Residency’ for birds wintering close to their breeding site on Franz-Josef Land, ‘Off Jan Mayen’ for birds migrating away from Franz-Josef Land to winter close to Jan Mayen in the Western North Atlantic, and ‘Pacific’ for birds engaging in transarctic migration from the North Atlantic into the North Pacific. In the latter case return migration from the Pacific towards the Atlantic might cross the central arctic basin, or follow the periphery. Since both return strategies induce similar costs (see results), we only present one data set. Error bars correspond to standard deviations capturing the variation between climatic models. Little auks drawings used in this figures were extracted from Richard Crossley’s picture (available online under CC-BY-SA license https://creativecommons.org/licenses/by-sa/2.0/legalcode at https://commons.wikimedia.org/wiki/File:Little_Auk_from_the_Crossley_ID_Guide_Britain_and_Ireland.jpg).

Little auks breeding in Franz Josef Land currently winter predominantly in the Barents Sea and off Jan Mayen (North Atlantic). In these areas, their current daily energy requirements are predicted to increase throughout the non-breeding period (September to March), from 449 +/− 57 to 760 +/− 6 kJ.day^−1^ off Jan Mayen and from 732 +/− 2 to 772 +/− 8 kJ.day^−1^ in the Barents Sea. Rising winter energy requirements has already been observed in the Atlantic for little auks overwintering off Newfoundland, and is explained by the decreasing air temperatures^48^. Across the winter period, birds were predicted to require a total of 138 +/− 3 MJ off Jan Mayen and 161 +/− 1 MJ in the Barents Sea. According to the four climatic models considered, winter energy requirements linked to the little auks’ current migratory strategy are predicted to decrease slightly in the future (Fig. 5.). Their future total energy requirements may therefore decrease to 119 +/− 4 MJ off Jan Mayen and to 158 +/− 0.1 MJ in the Barents Sea. In comparison, predicted daily requirements of little auks wintering in the North Pacific are considerably lower, and range, on average, from 267 +/− 2 kJ.day^−1^ in September to 323 +/− 6 kJ.day^−1^ or 322 +/− 5.8 kJ.day^−1^ in March according to the migratory flyway considered (direct or peripheral, respectively). Indeed, favorable thermal conditions encountered along the peripheral route offset the enhanced flight costs due to the greater travelling distance. Overall wintering costs (accounting for flights across the arctic basin) are 59 +/− 0.7 MJ for this transarctic strategy according to the four climatic models. Sea surface and air temperature are main drivers of little auk winter energy requirements^48,62^, and since those temperatures are higher in the North Pacific in winter, they explain lower overall energy requirements for little auks engaging in transarctic migration, despite higher flight costs. Little auks from Franz Josef Land are predicted to become year-round residents only under the ACCESS 1.3 and HadGEM2-ES climatic models: Under these conditions, their energy requirements are predicted to range between 737 +/− 2 kJ.day^−1^ in September and 761 +/− 2 kJ.day^−1^ in March. Little auk overall winter energy requirements for this residency strategy are then estimated to 159 +/− 0.3 MJ, similar to those of birds remaining in the Barents Sea in the future.

## Discussion

Our study is, to the best of our knowledge, the first to address the impact of global change on arctic seabird migratory ecology, focusing both on a multi-species synthesis and on detailed statistical and mechanistic modelling of eco-energetics in a spatial context. Crucially, our work strongly suggests that arctic cryosphere loss may overturn migration patterns from the Atlantic into the Pacific, at least in some species. Also, as a consequence of global warning, other species may stop migrating, to become year-round residents of the high-Arctic. Beyond these surprising results, our analyses provide a conceptual framework which may be useful to understand and predict future bird migration in other regions of the world.

### Potential limitations

Despite these advances, our results have limitations which should be examined carefully. First, even if we identified a suite of species for which migration may change radically in the near future, those remain a minority at the scale of the arctic seabird community. Selection criteria for future trans-arctic migrants or high-arctic residents were mainly morphological and physiological, and linked to their capacity to benefit from a sea ice free Arctic Ocean, and to feed on marine prey during the polar night. Thanks to new tracking technologies and winter expeditions, there is information available for some species^34,64^. Yet, the migratory biology and the nocturnal behavior of many arctic seabird species still has not been subjected to detailed work. As results from biotelemetry studies typically reveal unexpected animal performances^65,66^ we speculate that future investigations will lead to expanding the list of potential transarctic migrants or year-round high-arctic residents.

Second, a strong assumption of our modelling work is that migratory ecology is primarily driven by environmental factors. This ignores the evolutionary and cultural history of studied populations. Indeed, past distributions^3^, as well as local culture^67^ have been demonstrated to shape animal distributions and migratory pathways, beyond current biotic and abiotic forcing factors. Nevertheless, there is also strong evidence that migratory birds do adjust their migratory ecology following global change, even at small spatio-temporal scales^44^. Further, migratory divides occurring within populations, and sometimes even within the same pair of breeding adults, may lead conspecifics to display radically different migratory strategies, towards different ocean basins^68^.

Third, and along the same lines, we used species distribution models (SDM) and a mechanistic model (Niche Mapper), and our results are subjected to assumptions and limitations specific to these techniques^69–71^. The accuracy of Niche Mapper predictions has been discussed and rated positively^61^ and we will not reiterate this information here. With respect to SDMs, we assumed that little auks are (i) at equilibrium with their environment, (ii) that statistical links between bird distributions and environmental data will still hold in the future and that (iii) we characterized the whole Hutchinsonian ecological niche for this species. The little auk is a long-lived seabird with low fecundity and high adult inter-annual survival^72^, showing phenotypic plasticity^39^ at small temporal and spatial scales. Nevertheless, its sensitivity to environmental changes^39,41^ and the time scale chosen for our analysis allowed us to assume a steady state between little auks and their environment. Further, we also had to face some potential biases contained in the opportunistic occurrence data which we used, such as misidentification, geographical bias (data collected in places with easier access) and or/climatic bias (missing data from an area with different climatic characteristics). In our case, we reduced geographical and climatic biases impacts by choosing modelling procedures which minimize them when creating sets of pseudo-absences^54^.

Fourth, SDMs do not take into account biotic factors, such as trophic interactions, predation or competition^69^. This might explain why our model overfitted current distributions in Eastern Canada, notably by predicting suitable breeding habitat where little auks do not currently breed (with the exception of a single colony on Baffin Island). Such discrepancies might be explained by potential mismatches between seabird observed occurrences, biotic and abiotic factors. For example, shaping the suitability of future habitats, the availability of food will put strong constraints on future birds’ migration. By affecting the temperature, salinity, acidity and productivity of Arctic Ocean, sea ice melt will also drastically change the distribution of all marine taxa including fishes and zooplankton. Current and future prey fields are difficult to obtain at the scale considered, but should allow to better access the likelihood of future distribution and behavior of birds. Moreover, further information on rare but extreme events or on small scale conditions would be useful to increase our model performance when predicting suitable habitats: For example, if available, the presence of crevices for nesting would have been a practical factor to predict suitable breeding grounds and potentially avoid overfitting where strong slopes occur in the absence of scree.

Despite these limitations, SDMs presented in this study had high continuous Boyce indexes. Also, model outputs for current little auk distributions are in agreement with available bibliographic information^72,73^. For example, predicted suitable little auk breeding, foraging, and wintering habitats for the North Pacific are in agreement with the fact that individual little auks (typically less than five) are often observed on/near Saint Laurence Island in the Bering Sea^74^ but also near Japan^75,76^ or British Columbia^77^. Moreover, predicted current winter residency in Svalbard or South Greenland is also supported by observations of little auks off Spitsbergen during the polar night^34,35^. Finally, northwards shifts of suitable habitats predicted by our models are in agreement with others studies of marine top predators^78^ and on their prey^79,80^.

### From vagrancy to dispersal and migration

Beyond migration, seabird large-scale movements may also include vagrancy and dispersal^3^. These principles apply to all organisms on the move but, to remain in an arctic context, we will illustrate them using our case study of little auks. In this species, vagrants (as defined by Newton^3^) may leave North Atlantic breeding colonies, to fly across the Arctic Basin and reach the North Pacific, but without breeding there or ever returning to the Atlantic. Dispersing individuals (sensu Clobert *et al*.^81^) may show the same behavior, but are predicted to settle and breed (or at least attempt to) in the North Pacific. This might well be the case for the very few little auk individuals which are sighted on St Lawrence Island in the Bering Sea^74^. Under current and near future sea ice conditions, vagrancy and dispersal into the North Pacific are more likely to occur in little auks, than complete migration as hypothesized in our study. Indeed, in the case of a full migration between a North Atlantic breeding site and the North Pacific, the returning journey in spring will have to be peripheral to avoid dense sea ice and the presence and quality of future stop-over sites (as polynyas) will be major constraints. Indeed, polynyas have long been established as key feeding and resting sites for a wide range of polar organisms^82–84^, especially during the winter period. Where and when polynyas will occur in the arctic in the future is nonetheless difficult to predict, but they are predicted to be impacted negatively by global warming^85^. Vagrants, and dispersing individuals, which do not travel back to the Atlantic, will not be affected by spring sea ice conditions in the Arctic Basin, and are therefore more likely to engage in a one way transarctic flight to the North Pacific. Finally, those movements to the opposite side of the Arctic could lead to genetic mixing between previously-isolated populations, and encourage transmission of diseases/parasites.

### Global relevance

The Arctic is subjected to drastic environmental changes and, at the request of arctic peoples, there is much research on the fate of species emblematic to this vast region, including birds (see^86^). Understanding current and future arctic bird distribution and migration has therefore been identified as a key objective by the Arctic Council and its working groups (in particular through the AMBI project https://www.caff.is/arctic-migratory-birds-initiative-ambi) and are the aim of recent studies^45,62,87,88^. With sea ice melt, the Arctic will be more and more exposed to human pressures such as gas/oil extraction, fisheries, marine traffic or tourism. Detailed ecological knowledge is therefore essential for the design of adaptive conservation strategies, within advanced marine spatial planning^45,89,90^. Marine Protected Areas (see http://www.mpatlas.org/ for detailed maps) already exist in the Arctic, but are lacking in some key areas such as the Bering Sea or along the Northern Canadian coast. Even though our conclusions have to be taken with all necessary caution, as detailed across the previous sections, our work suggests that arctic bird distribution and migratory pathways may shift radically within the next few decades. Overall, the establishment of future Marine Protected Area have to evolve with those shifts, preserving wintering and breeding grounds but also stop-over sites needed by the vast majority of migratory arctic birds. The modalities and likelihood of forthcoming major changes will thereby be investigated, both theoretically when studying migration ecology^91^, during winter field expeditions^34^ and via biotelemetry studies^92,93^.

On a worldwide scale, we speculate that other migratory pathways may be shifted by global change. Notably, there are strong signals that Pacific seabirds may also migrate into the Atlantic via the North Pole^31^. Transcontinental bird migrations currently occur on eight flyways which all run North-South along the Americas, Africa-Eurasia, and Australasia^94^. Whether populations of migratory birds using these flyways will go extinct following global change impacts, or will radically shift migratory pathways and/or strategies, will be the subject of some exciting research in the near-future

## Supplementary information

Supplementary materials

## Data Availability

Data used in this study are in open access on the respective providers’ website (see Materials and Methods) excepting occurrence data from Norwegian Polar Institute (Strøm *et al*., 2008).
