# Supplementary material for: Climate change could overturn bird migration: Transarctic flights and high-latitude residency in a sea ice free Arctic
Source: Sci Rep. 2019 Nov 28;9:17767. doi: 10.1038/s41598-019-54228-5 (PMC6883031; doi:10.1038/s41598-019-54228-5)

Climate change could overturn bird migration: Transarctic flights and high-latitude residency in a sea ice free Arctic

Manon Clairbaux[1*], Jérôme Fort[2], Paul Mathewson[3], Warren Porter[3], Hallvard Strøm[4], David Grémillet[1,5]

[1]CEFE UMR 5175, CNRS – Université de Montpellier – Université Paul-Valéry Montpellier – EPHE, Montpellier, France

[2]Littoral Environnement et Sociétés (LIENSs), UMR 7266 CNRS-Université de La Rochelle, La Rochelle, France

[3]Department of Integrative Biology, University of Wisconsin, Madison, WI, USA

[4]Norwegian Polar Institute, Fram Centre, P.O. Box 6606 Langnes, 9296 Tromsø, Norway

[5]Percy FitzPatrick Institute, DST/NRF Centre of Excellence, University of Cape Town, Rondebosch, South Africa

* Corresponding author, manon.clairbaux@cefe.cnrs.fr or 04.67.61.33.09 (FRANCE)

Supplemental materials I: Current potential distribution of little auks (*Alle alle*) and occurrence data points

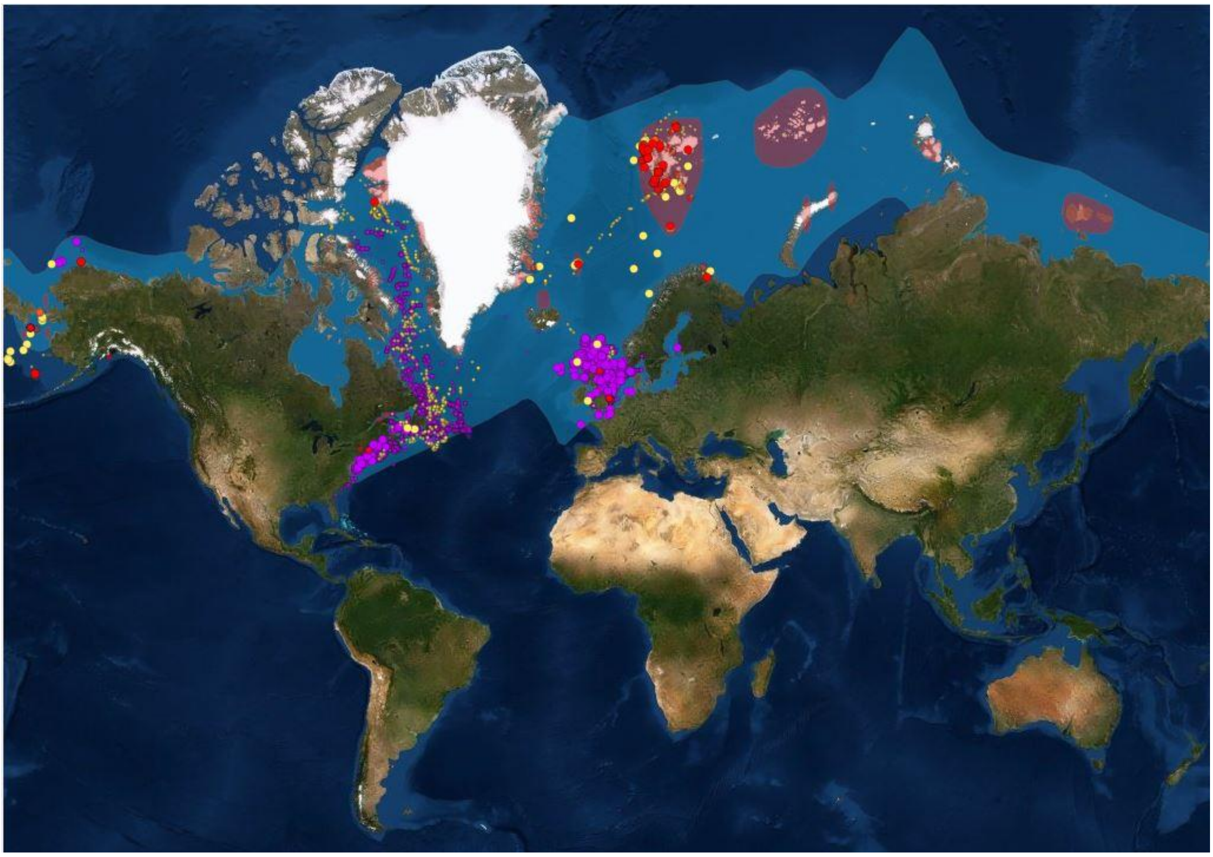

This map has been created using ESRI ArcGis Online with satellite imagery from EarthStar Geographics. Sources: Esri, DigitalGlobe, Earthstar Geographics, CNES/Airbus DS, GeoEye, USDA FSA, USGS, Getmapping, Aerogrid, IGN, IGP, and the GIS User Community.

The current potential distribution of little auks was digitalized by the GROMS (Global Register of Migratory Species) from "Handbook of the Birds of the World. Vol.3 Hoatzin to Auks" by del Hoyo, Elliot and Sargatal (1996). Red patches are areas potentially used by Little auks during breeding and the blue region is potentially used during foraging or wintering.

Red, yellow and purple dots are the occurrence data points available respectively for breeding periods, foraging during summer and wintering periods. Larger dots are those used while modelling current little auks distribution. Smaller dots represent positions which were excluded from the analysis because some associated environmental variables were lacking.

Supplemental materials II: Summary of parameters used in Niche Mapper ™

Morphological properties	Value	References
Body mass (g)	202	Stempniewicz et al., 2016
Body plumage depth (dorsal-ventral) (mm)	7.4-12.8	Fort et al., 2009
Head plumage depth (d-v) (mm)	5.2-9.4	Amélineau et al., 2018
Plumage reflectivity (d-v) (%)	40.4-65.0	Fort et al., 2009
Body feather length (d-v) (mm)	20.0-19.2	Fort et al., 2009
Head feather length (d-v) (mm)	11.6-10.7	Amélineau et al., 2018
Feather diameter (d-v) (µm)	33.0-33.0	Fort et al., 2009
**Physiological properties**		
Body core temperature (°C)	40	Gabrielsen et al., 1991
Flesh thermal conductivity ($W.m^{-1}.°C^{-1}$)	0.4-2.8	Cheng & Plewes, 1992
Oxygen extraction efficiency (%)	35	Arens & Cooper, 2005
Bird density ($kg.m^{-3}$)	932.9	Porter et al., 2006
Resting metabolic rate (W)	2.02	Gabrielsen et al., 1991
Flight metabolism (W)	12.9	Raveling & LeFebvre, 1967
Diving metabolism (W)	2.5*Resting metabolic rate	Croll & McLaren, 1993
**Behavioural properties**		
Ventral area contacting substrate (%)	25	Fort et al., 2009
Proportion of time spent flying per day during winter (%)	9	Fort et al., 2009,2010
Proportion of time spent flying per day during migratory flight (%) according migratory strategy	$\frac{\text{Distance between colony and wintering site} * 100}{\text{Flight speed} * 86400 * 30}$	This study
Proportion of time spent diving per day (%)	24	Fort et al.,2010
**Environmental data**		
Sea surface temperature (°C)		Climatic models previously used according time period (2000-2017 or 2050-2059) and RCP8.5 scenario
Air temperature (°C)		
Cloud over (%)	0-100	
Relative humidity (%)		
Wind speed ($m.s^{-1}$)		

Little auk is modelled by several ellipsoids (head, body and legs) as a multipart animal (Amélineau et al., 2018) in the animal module of Niche Mapper ™. When not diving or flying, little auk is modelled as resting at the surface of water, with 25 % of its body in contact with water: the model targeted the resting metabolic rate for all hours except for the scheduled diving and flight hours.

Supplemental materials III: List of migratory/partial migratory species to which the Arctic region represents more than 5% of their breeding range.

Families, Latin names and code for winter's habitats are those used in CAFF's technical report (Scott, 1998).

Code for winter's habitats:

PE: Pelagic (Open Ocean)

CM: Coastal marine habitats (inshore marine waters)

CW: Coastal wetlands (mudflats, sandy beaches, rocky shores, mangroves)

IW: Freshwater and blackish wetlands, including deep water lakes

OC: Open country habitats

GR: Grasslands, steppe and arable land

DE: Desert habitats

DF: Temperate forests and woodland (coniferous and deciduous)

TF: Tropical forests (lowland and montane)

TW: Tropical woodland (acacia woodland, parkland, scrub)

Families	Common names	Latin names	Wintering habitat
**Gaviidae**	Red-throated diver	*Gavia stellata*	CM
	Black-throated diver	*Gavia arctica*	CM
	Pacific loon	*Gavia pacifica*	CM
	Great northern diver	*Gavia immer*	CM
	White billed diver	*Gavia adamsii*	CM
**Podicipedidae**	Red-necked grebe	*Podiceps grisegena*	CM
	Horned grebe	*Podiceps auritus*	CM
**Procellariidae**	**Northern fulmar**	***Fulmarus glacialis***	**PE**
	**Manx shearwater**	***Puffinus puffinus***	**PE**
**Hydrobatidae**	**European storm petrel**	***Hydrobates pelagicus***	**PE**
	**Leach's storm-petrel**	***Oceanodroma leucorhoa***	**PE**
	**Fork-tailed storm-petrel**	***Oceanodroma furcata***	**PE**
**Sulidae**	**Northern gannet**	***Morus bassanus***	**PE**
**Phalacrocoracidae**	Double-crested cormorant	*Phalacrocorax auritus*	CM
	Great cormorant	*Phalacrocorax carbo*	CM
	Red-faced cormorant	*Phalacrocorax urile*	CM
	Pelagic cormorant	*Phalacrocorax pelagicus*	CM
	European shag	*Phalacrocorax aristotelis*	CM

Anatidae	Whooper swan	*Cygnus cygnus*	IW
	Tundra swan	*Cygnus columbianus*	IW
	Pink-footed goose	*Anser brachyrhynchus*	GR
	Taiga bean goose	*Anser fabalis*	GR
	Greater white-fronted goose	*Anser albifrons*	GR
	Lesser white-fronted goose	*Anser erythropus*	GR
	Greylag goose	*Anser anser*	GR
	Snow goose	*Anser caerulescens*	GR
	Ross goose	*Anser rossii*	GR
	Emperor goose	*Anser canagica*	CW
	Canada goose	*Branta canadensis*	GR
	Barnacle goose	*Branta leucopsis*	GR
	Brent goose	*Branta bernicla*	CW
	Red-breasted goose	*Branta ruficollis*	GR
	Eurasian wigeon	*Anas penelope*	IW
	American wigeon	*Anas americana*	IW
	Baikal teal	*Anas formosa*	IW
	Common teal	*Anas crecca*	IW
	Mallard	*Anas platyrynchos*	IW
	American black duck	*Anas rubripes*	IW
	Northern pintail	*Anas acuta*	IW
	Northern shoveler	*Anas clypeata*	IW
	Canvasback	*Aythya valisineria*	IW
	Tufted duck	*Aythya fuligula*	IW
	Greater scaup	*Aythya marila*	CM
	Lesser Scaup	*Aythia affinis*	IW
	Common eider	*Somateria mollissima*	CM
	King eider	*Somateria spectabilis*	CM
	Spectacled eider	*Somateria fischeri*	CM
	Steller's eider	*Polysticta stelleri*	CM
	Harlequin duck	*Histrionicus histrionicus*	CM
	Long tailed duck	*Clangula hyemalis*	CM
	Black scoter	*Melanitta nigra*	CM
	Surf scoter	*Melanitta perspicillata*	CM
	White-winged scoter	*Melanitta fusca*	CM
	Common goldeneye	*Bucephala clangula*	IW
	Barrow's Goldeneye	*Bucephala islandica*	CM
	Smew	*Mergellus albellus*	IW
	Red-brested merganser	*Mergus serrator*	CM

	Goosander	*Mergus merganser*	IW
**Pandionidae**	Western osprey	*Pandion haliaetus*	IW
**Accipitridae**	White-tailed eagle	*Haliaeetus albicilla*	IW
	Bald eagle	*Haliaeetus leucocephalus*	IW
	Steller's sea eagle	*Haliaeetus pelagicus*	CW
	Hen harrier	*Circus cyaneus*	IW
	Eurasian sparrowhawk	*Accipiter nisus*	DF
	Sharp-shinned hawk	*Accipiter striatus*	DF
	Northern goshawk	*Accipiter gentilis*	DF
	Swainson' hawk	*Buteo swainsoni*	OC
	Red-tailed hawk	*Buteo jamaicensis*	OC
	Rough-legged buzzard	*Buteo lagopus*	OC
	Golden eagle	*Aquila chrysaetos*	OC
**Falconidae**	Common kestrel	*Falco tinnunculus*	OC
	American kestrel	*Falco sparverius*	OC
	Merlin	*Falco columbarius*	OC
	Gyrfalcon	*Falco rusticolus*	OC
	Peregrine falcon	*Falco peregrinus*	OC
**Gruidae**	Siberian crane	*Grus leucogeranus*	IW
	Sandhill crane	*Grus canadensis*	GR
	Common crane	*Grus grus*	GR
	Whooping crane	*Grus americana*	IW
**Rallidae**	Yellow rail	*Coturnicops noveboracensis*	IW
	Sora	*Porzana carolina*	IW
**Haematopodidae**	Eurasian oystercatcher	*Haematopus ostralegus*	CW
**Charadriidae**	European golden plover	*Pluvialis apricaria*	GR
	Pacific golden plover	*Pluvialis fulva*	GR
	American golden plover	*Pluvialis dominica*	GR
	Grey plover	*Pluvialis squatarola*	CW
	Common ringed plover	*Charadrius hiaticula*	CW
	Semipalmated plover	*Charadrius semipalmatus*	CW
	Killdeer	*Charadrius vociferus*	GR
	Lesser sandplover	*Charadrius mongolus*	CW
	Eurasian dotterel	*Eudromias morinellus*	DE
	Northern lapwing	*Vanellus vanellus*	GR
**Scolopacidae**	Eurasian woodcok	*Scolopax rusticola*	DF
	Pin-tailed snipe	*Gallinago stenura*	IW
	Great snipe	*Gallinago media*	IW
	Common snipe	*Gallinago gallinago*	IW

	Jack snipe	*Lymnocryptes minimus*	IW
	Black-tailed godwit	*Limosa limosa*	CW
	Hudsonian godwit	*Limosa haemastica*	CW
	Bar-tailed godwit	*Limosa lapponica*	CW
	Little curlew	*Numenius minutus*	GR
	Eskimo curlew	*Numenius borealis*	GR
	Whimbrel	*Numenius phaeopus*	CW
	Bristle-thighed curlew	*Numenius tahitiensis*	CW
	Eurasian curlew	*Numenius arquata*	CW
	Spotted redshank	*Tringa erythropus*	IW
	Common redshank	*Tringa totanus*	CW
	Common greenshank	*Tringa nebularia*	IW
	Greater yellowlegs	*Tringa melanoleuca*	IW
	Lesser yellowlegs	*Tringa flavipes*	IW
	Solitary sandpiper	*Tringa solitaria*	IW
	Green sandpiper	*Tringa ochropus*	IW
	Wood sandpiper	*Tringa glareola*	IW
	Terek sandpiper	*Tringa cinereus*	CW
	Common sandpiper	*Tringa hypoleucos*	IW
	Spotted sandpiper	*Tringa macularia*	IW
	Grey-tailed tattler	*Tringa brevipes*	CW
	Wandering tattler	*Tringa incanus*	CW
	Ruddy turnstone	*Arenaria interpres*	CW
	Black turnstone	*Arenaria melanocephala*	CW
	Short-billed dowitcher	*Limnodromus griseus*	CW
	Long billed dowitcher	*Limnodromus scolopaceus*	CW
	Surfbird	*Aphriza virgata*	CW
	Great knot	*Calidris tenuirostris*	CW
	Red knot	*Calidris canutus*	CW
	Sanderling	*Calidris alba*	CW
	Semipalmated sandpiper	*Calidris pusilla*	CW
	Western sandpiper	*Calidris mauri*	CW
	Little stint	*Calidirs minuta*	CW
	Red-necked stint	*Calidris ruficollis*	CW
	Temminck's stint	*Calidris temminckii*	IW
	Long toed stint	*Calidris subminuta*	IW
	Least sandpiper	*Calidris minutilla*	IW
	White rumped sandpiper	*Calidris fuscicollis*	CW
	Baird's sandpiper	*Calidris bairdii*	IW

	Pectoral sandpiper	*Calidris melanolos*	IW
	Sharp-tailed sandpiper	*Calidris acuminata*	IW
	Purple sandpiper	*Calidris maritima*	CW
	Rock sandpiper	*Calidris ptilocnemis*	CW
	Dunlin	*Calidris alpina*	CW
	Curlew sandpiper	*Calidris ferruginea*	CW
	Stilt sandpiper	*Micropalama himantopus*	IW
	Buff-breasted sandpiper	*Tryngites subruficollis*	GR
	Spoon billed sandpiper	*Eurynorhynchus pygmeus*	CW
	Broad billed sandpiper	*Limicola falcinellus*	CW
	Ruff	*Philomachus pugnax*	IW
	**Red-necked phalarope**	***Phalaropus lobatus***	**PE**
	**Red phalarope**	***Phalaropus fulicaria***	**PE**
**Stercorariidae**	**Great skua**	***Catharacta skua***	**PE**
	**Pomarine skua**	***Stercorarius Pomarinus***	**PE**
	**Arctic skua**	***Stercorarius parasiticus***	**PE**
	**Long tailed skua**	***Stercorarius longicaudus***	**PE**
**Laridae**	Mew gull	*Larus canus*	CW
	Larus californicus	*Larus californicus*	CW
	Great black backed gull	*Larus marinus*	CM
	Glaucus winged gull	*Larus glaucescens*	CM
	Glaucous gull	*Larus hyperboreus*	CM
	Iceland gull	*Larus glaucoides*	CM
	Herring gull	*Larus argentatus*	CM
	Lesser black backed gull	*Larus fuscus*	CM
	Common back headed gull	*Larus ridibundus*	CW
	Bonaparte's gull	*Larus philadelphia*	CW
	**Ivory gull**	***Pagophila eburnea***	**PE**
	**Ross's gull**	***Rhodostethia rosea***	**PE**
	**Sabine's gull**	***Xerna sabini***	**PE**
	**Black legged kittiwake**	***Rissa tridactyla***	**PE**
	**Red legged kittiwake**	***Rissa brevirostris***	**PE**
	Caspian tern	*Sterna caspia*	CM
	Common tern	*Sterna hirundo*	CM
	**Arctic tern**	***Sterna paradisaea***	**PE**
	**Aleutian tern**	***Sterna aleutica***	**PE**
**Alcidae**	**Little auk**	***Alle alle***	**PE**
	**Common murre**	***Uria aalge***	**PE**
	**Thick billed murre**	***Uria lomvia***	**PE**

	Razorbill	*Alca torda*	CM
	Marbled murrelet	*Brachyramphus marmoratus*	CM
	Kittlitz'smurrelet	*Brachyramphus brevirostris*	CM
	**Ancient murrelet**	***Synthliboramphus antiquus***	**PE**
	**Cassin's auklet**	***Piychoramphus aleuticus***	**PE**
	Parakeet auklet	*Cyclorrhynchus psittacula*	CM
	Crested auklet	*Aethia cristatella*	CM
	**Whiskered auklet**	***Aethia pygmaea***	**PE**
	**Least auklet**	***Aethia pusilla***	**PE**
	**Atlantic puffin**	***Fratercula arctica***	**PE**
	**Horned puffin**	***Fratercula corniculata***	**PE**
	**Tufted puffin**	***Fratercula cirrhata***	**PE**
**Cuculidae**	Common cuckoo	*Cuculus canorus*	TW
	Himalayan cuckoo	*Cuculus saturatus*	TF
**Strigidae**	Snowy owl	*Nyctea scandiaca*	OC
	Boreal owl	*Aegolius funereus*	DF
	Long-eared owl	*Asio otus*	DF
	Short-eared owl	*Asio flammeus*	OC
**Caprimulgidae**	Common nightawk	*Chordeiles minor*	OC
**Apopidae**	Pacific swift	*Apus pacificus*	OC
**Alcedinidae**	Belted kingfisher	*Ceryle alcyon*	IW
**Picidae**	Eurasian wryneck	*Jynx torquilla*	TW
	Yellow-belled sapsucker	*Sphyrapicus varius*	DF
	Hairy woodpecker	*Picoides villosus*	DF
	Northern flicker	*Colaptes auratus*	DF
**Tyrannidae**	Olive-sided flycatcher	*Contopus borealis*	TF
	Western wood pewee	*Contopus sordidulus*	TF
	Yellow-bellied flycatcher	*Empidonax flaviventris*	TF
	Alder flycatcher	*Empidonax alnorum*	TF
	Least flycatcher	*Empidonax minimus*	TF
	Eastern phoebe	*Sayornis phoebe*	OC
	Say's phoebe	*Sayornis saya*	OC
	Eastern kingbird	*Tyrannus tyrannus*	OC
**Alaudidae**	Eurasian skylarck	*Alauda arvensis*	GR
	Horned lark	*Eremophila alpestris*	GR
**Hirundinidae**	Tree swallow	*Tachycineta bicolor*	OC
	Violet green swallow	*Tachycineta thalassina*	OC
	Sand martin	*Riparia riparia*	IW

	Barn swallow	*Hirundo rustica*	OC
	Cliff swallow	*Hirundo pyrrhonota*	OC
	House martin	*Delichon urbica*	TF
**Motocillidae**	White wagtail	*Motacilla alba*	OC
	Citrine wagtail	*Motacilla citreola*	IW
	Western yellow wagtail	*Motacilla flava*	GR
	Tree pipit	*Anthus trivialis*	TW
	Olive-backed pipit	*Anthus hodgsoni*	TW
	Pechora pipit	*Anthus gustavi*	TF
	Meadow pipit	*Anthus pratensis*	GR
	Red-throated pipit	*Anthus cervinus*	IW
	Eurasian Rock Pipit	*Anthus petrosus*	CW
	Buff bellied pipit	*Anthus rubescens*	IW
**Laniidae**	Brown shrike	*Lanius cristatus*	OC
	Great grey shrike	*Lanius excubitor*	OC
**Bombycillidae**	Bohemian waxwing	*Bombycilla garrulus*	DF
**Cinclidae**	White-troated dipper	*Cinclus cinclus*	IW
**Troglodytidae**	Eurasian wren	*Troglodytes troglodytes*	DF
**Prunellidae**	Siberian accentor	*Prunella montanella*	DF
	Dunnock	*Prunella modularis*	DF
**Muscicapidae-Turdinae**	Varied trush	*Zoothera naevia*	DF
	Grey-cheecked trush	*Catharus minimus*	TF
	Swainson's trush	*Catharus ustulatus*	TF
	Hermit trush	*Catharus guttatus*	TF
	Ring ouzel	*Turdus torquatus*	OC
	Common blackbird	*Turdus merula*	DF
	Eyebrowed trush	*Turdus obscurus*	TF
	Naumann's trush	*Turdus naumanni*	DF
	Fielfare	*Turdus pilaris*	DF
	Redwing	*Turdus iliacus*	DF
	Song trush	*Turdus philomelos*	DF
	Mistle trush	*Turdus viscivorus*	DF
	American robin	*Turdus migratorius*	DF
	European robin	*Erithacus rubecula*	DF
	Siberian rubythroat	*Luscinia calliope*	TW
	Bluethroat	*Luscinia svecica*	IW
	Red-flanked bluetail	*Tarsiger cyanurus*	TF
	Common redstart	*Phoenicurus phoenicurus*	TW
	Whinchat	*Saxicola rubetra*	TW

	Siberian stonechat	*Saxicola maura*	OC
	Northern wheatear	*Oenanthe oenanthe*	OC
**Muscicapidae-sylviinae**	Lanceolated Warbler	*Locustella lanceolata*	GR
	Sedge warbler	*Acrocephalus schoenobaenus*	IW
	Willow warbler	*Phylloscopus trochilus*	TW
	Common chiffchaff	*Phylloscopus collybita*	TW
	Yellow-browed warbler	*Phylloscopus inornatus*	TF
	Arctic warbler	*Phylloscopus borealis*	TF
	Eurasian blackcap	*Sylvia atricapilla*	TW
	Garden warbler	*Sylvia borin*	TW
	Rugy-crowned kinglet	*Regulus calendula*	DF
	Goldcrest	*Regulus regulus*	DF
	Golden-crowned kinglet	*Regulus satrapa*	DF
**Muscicapidae-Muscicapinae**	Spotted flycatcher	*Muscicapa striata*	TW
	European pied flycatcher	*Ficedula hypoleuca*	TF
	Red-breasted flycatcher	*Ficedula parva*	TF
**Certhiidae**	Eurasian treecreeper	*Certhia familiaris*	DF
	Brown creeper	*Certhia americana*	DF
**Emberizidae-Emberizinae**	Yellowhammer	*Emberiza citrinella*	GR
	Little bunting	*Emberiza pusilla*	OC
	Rustic bunting	*Emberiza rustica*	DF
	Yellow-breasted bunting	*Emberiza aureola*	GR
	Pallas's reed bunting	*Emberiza pallasi*	IW
	Common reed bunting	*Emberiza schoeniclus*	IW
	Lapland longspur	*Calcarius lapponicus*	GR
	Smith's longspur	*Calcarius pictus*	GR
	Snow bunting	*Plectrophenax nivalis*	OC
	McKay's bunting	*Plectrophenax hyperboreus*	OC
	Red fox sparrow	*Passerella iliaca*	DF
	Song sparrow	*Melospiza melodia*	DF
	Lincoln's sparrow	*Melospiza lincolnii*	IW
	Swamps sparrow	*Melospiza georgiana*	IW
	Harris's sparrow	*Zonotrichia querula*	DF
	White-crowned sparrow	*Zonotrichia leucophrys*	DF
	White throated sparrow	*Zonotrichia albicollis*	DF
	Golden-crowned sparrow	*Zonotrichia atricapilla*	DF
	Dark-eyed junco	*Junco hyemalis*	DF
	Savannah sparrow	*Passerculus sandwichensis*	GR

	American tree sparrow	*Spizella arborea*	OC
	Chipping sparrow	*Spizella passerina*	GR
**Parulidae**	Tennessee warbler	*Vermivora peregrina*	TF
	Orange-crowned warbler	*Vermivora celata*	TF
	American yellow warbler	*Dendroica petechia*	TF
	Yellow-rumped warbler	*Dendroica coronata*	DF
	Blackpoll warbler	*Dendroica striata*	TF
	Black and white warbler	*Mniotilta varia*	TF
	Northern waterthrush	*Seiurus noveboracensis*	IW
	Wilson's warbler	*Wilsonia pusilla*	TF
**Vireonidae**	Red-eyed vireo	*Vireo olivaceus*	TF
	Warbling vireo	*Vireo gilvus*	TF
**Icteridae**	Red-winged blackbird	*Agelaius phoeniceus*	IW
	Rusty blackbird	*Euphagus carolinus*	DF
**Fringillidae**	Brambling	*Fringilla montifringilla*	DF
	Pine siskin	*Carduelis pinus*	DF
	Arctic redpoll	*Carduelis hornemanni*	DF
	Common redpoll	*Carduelis flammea*	DF
	Twite	*Carduelis flavirostris*	OC
	Asian rosy finch	*Leucosticte arctoa*	OC
	Common rosefinch	*Carpodacus erythrinus*	TF
	Purple finch	*Carpodacus purpureus*	DF
	Pine grosbeak	*Pinicola enucleator*	DF
	Red crossbill	*Loxia curvirostra*	DF
**Sturnidae**	Common starling	*Sturnus vulgaris*	OC
**Corvidae**	American crow	*Corvus brachyrhynchos*	OC
	Carrion crow	*Corvus corone*	OC

Supplemental materials IV: List of coastal/marine bird species likely to be able to cope with the polar night according to bibliographic information

Families, Latin names and code for winter's habitats are those used in CAFF's technical report (Scott, 1998)

Families	Common names	Latin names	References about nocturnal activity
**Gaviidae**	White billed diver	*Gavia adamsii*	Earnst, 2004
**Procellariidae**	Northern fulmar	*Fulmarus glacialis*	Berge et al., 2012
	Manx shearwater	*Puffinus puffinus*	Martin et al.,1991
**Hydrobatidae**	European storm petrel	*Hydrobates pelagicus*	del Hoyo et al., 1996
	Leach's storm-petrel	*Oceanodroma leucorhoa*	
	Fork-tailed storm-petrel	*Oceanodroma furcata*	
**Phalacrocoracidae**	Double-crested cormorant	*Phalacrocorax auritus*	King et al., 1998
	Great cormorant	*Phalacrocorax carbo*	Grémillet, 2005
**Anatidae**	Greater scaup	*Aythya marila*	Beynon et al., 1981
	Common eider	*Somateria mollissima*	Merkel et al., 2008; Merkel & Mosbech, 2007
	Steller's eider	*Polysticta stelleri*	Systad & Bustnes, 2001
**Scolopacidae**	Red phalarope	*Phalaropus fulicaria*	McNeil et al., 1992
**Stercorariidae**	Great skua	*Catharacta skua*	Votier et al., 2006
**Laridae**	Great black backed gull	*Larus marinus*	Garthe & Huppop, 1996
	Lesser black backed gull	*Larus fuscus*	
	Ivory gull	*Pagophila eburnea*	Defenders of wildlife, 2007
	Black legged kittiwake	*Rissa tridactyla*	Berge et al., 2012
	Red legged kittiwake	*Rissa brevirostris*	Storer, 1987; Orben et al.,2014
**Alcidae**	Little auk	*Alle alle*	Berge et al., 2015; Ostaszewsha et al., 2017
	Common murre	*Uria aalge*	Regular et al., 2011
	Thick billed murre	*Uria lomvia*	Regular et al., 2011
	Razorbill	*Alca torda*	Bienvenuti et al., 2001
	Cassin's auklet	*Pachoramphus aleuticus*	Nelson, 1989
	Whiskered auklet	*Aethia pygmaea*	Zubakin & Konyukhov, 1999

Supplemental materials V: Mapping current and future projection of little auks' habitats according to climatic models

Graticules are set at a 15° interval and the map is projected as North Pole Lambert Azimuthal Equal Area. This map and the followings have been made using R software (version 3.5.1, https://cran.r-project.org/) thanks "maptools","rgdal","rgeos" and "sp" packages.

Current projection

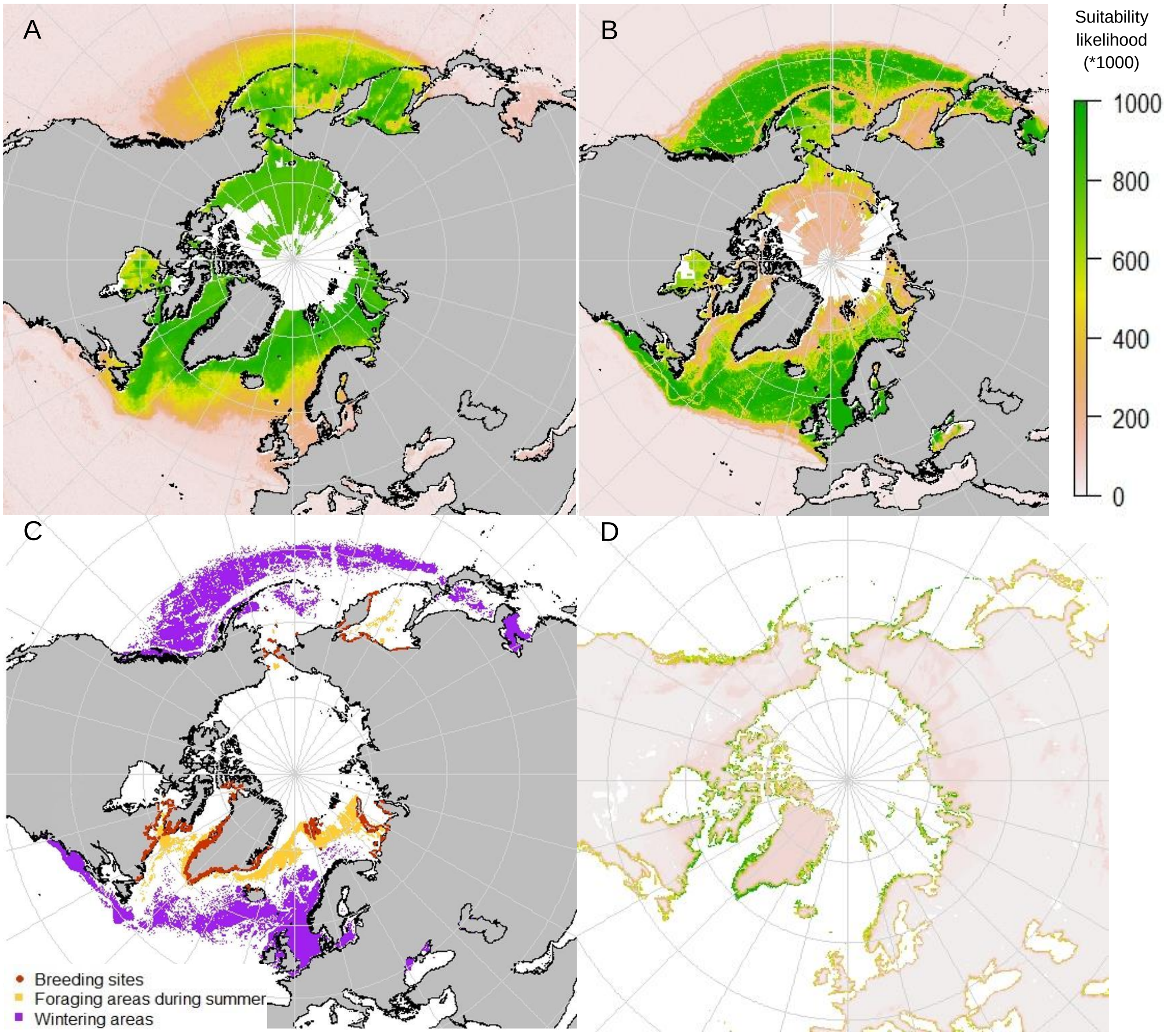

Projection of suitability probability (*1000) between 2000-2017 for foraging during summer (figure A), winter (figure B) and breeding (figure D). Suitable habitats (suitability likelihood >0.9) for those periods are shown in figure C.

In the following parts, projection of suitability probability (*1000) between 2050-2059 for foraging during summer (figure A), winter (figure B) and breeding (figure D) obtained with the four climatic models within the RCP8.5 scenario are presented. Suitable habitats (suitability likelihood >0.9) for those periods are shown in figure C. Changes in the distribution of suitable foraging (figure E) and wintering (figure F) areas between present (2000-2017) and future (2050-2059, climatic model considered, scenario RCP 8.5) have been deduced comparing projection output.

Future projection with the HadGEM2-CC climatic model

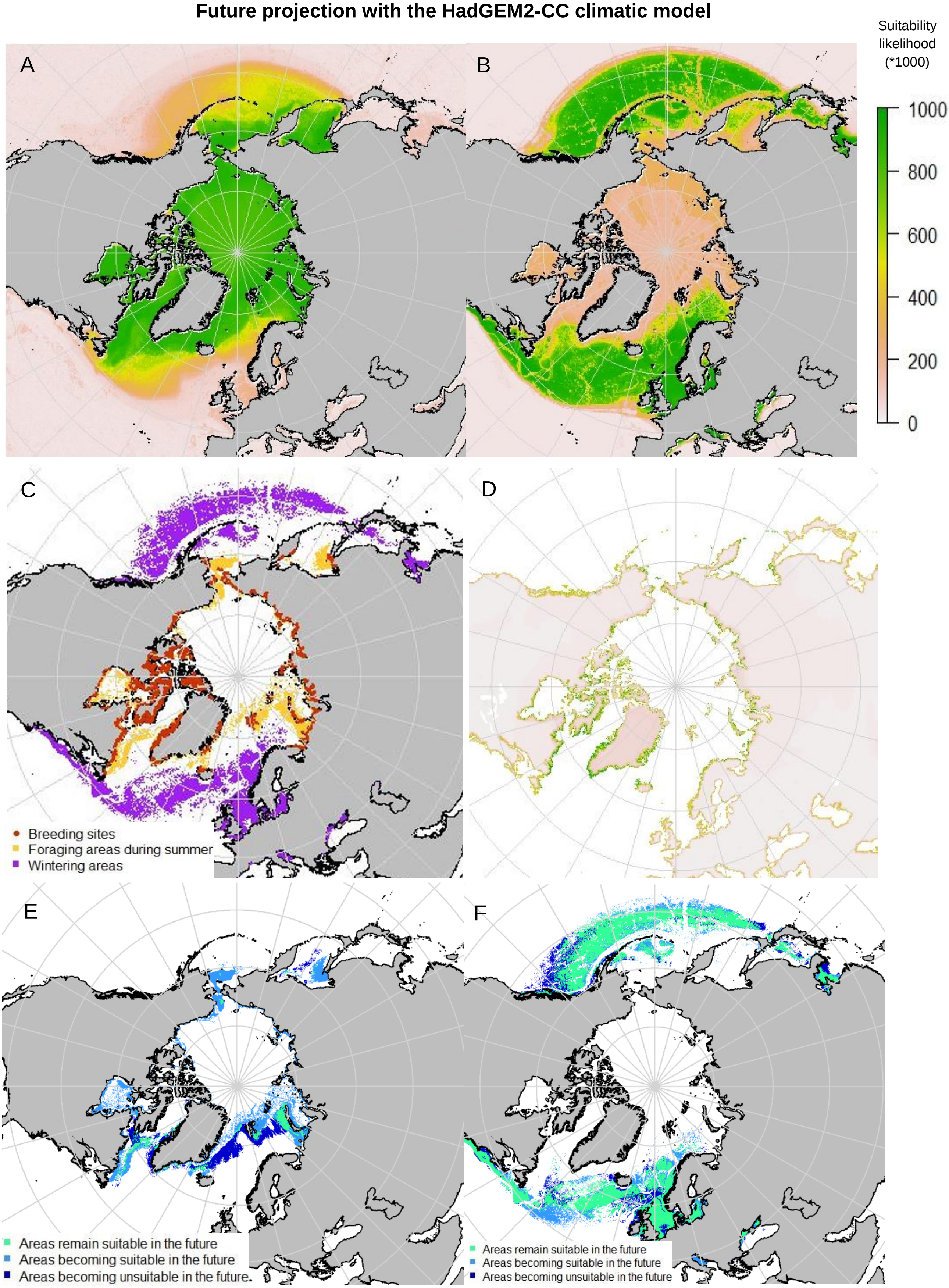

Future projection with the HadGEM2-ES climatic model

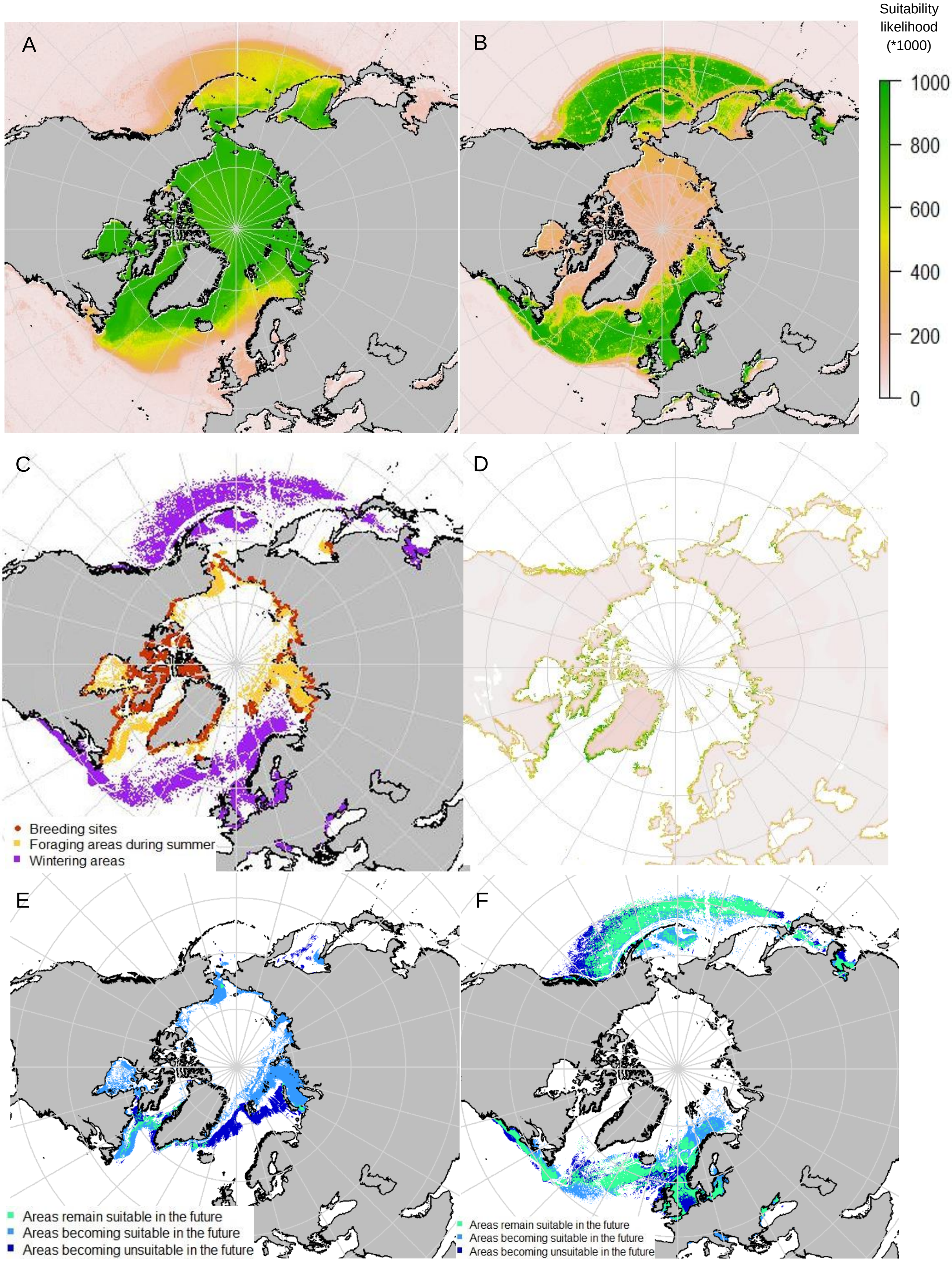

Future projection with the ACCESS 1.0 climatic model

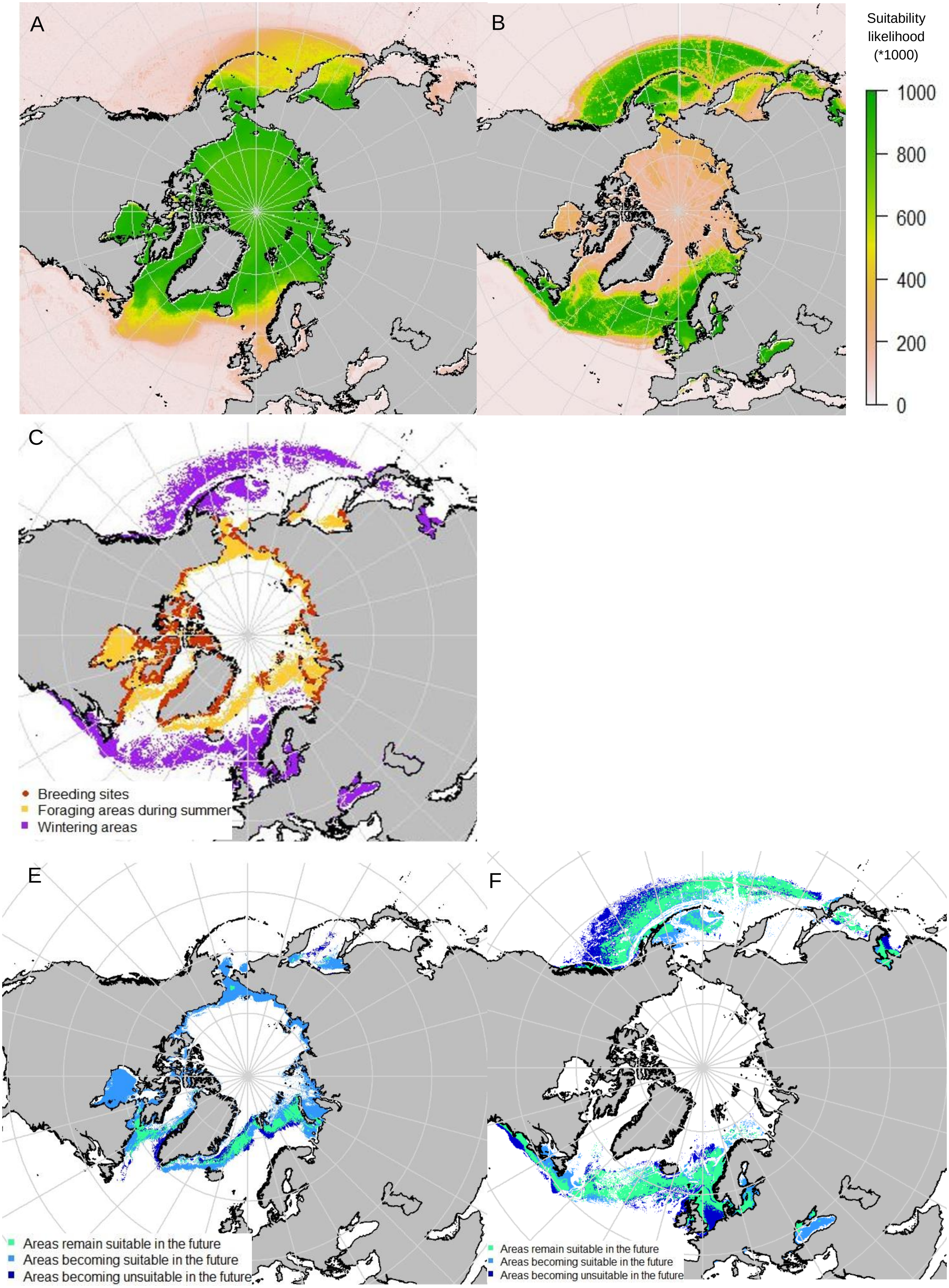

Breeding sites are those obtained with the HadGEM models (figure D is missing).

Future projection with the ACCESS 1.3 climatic model

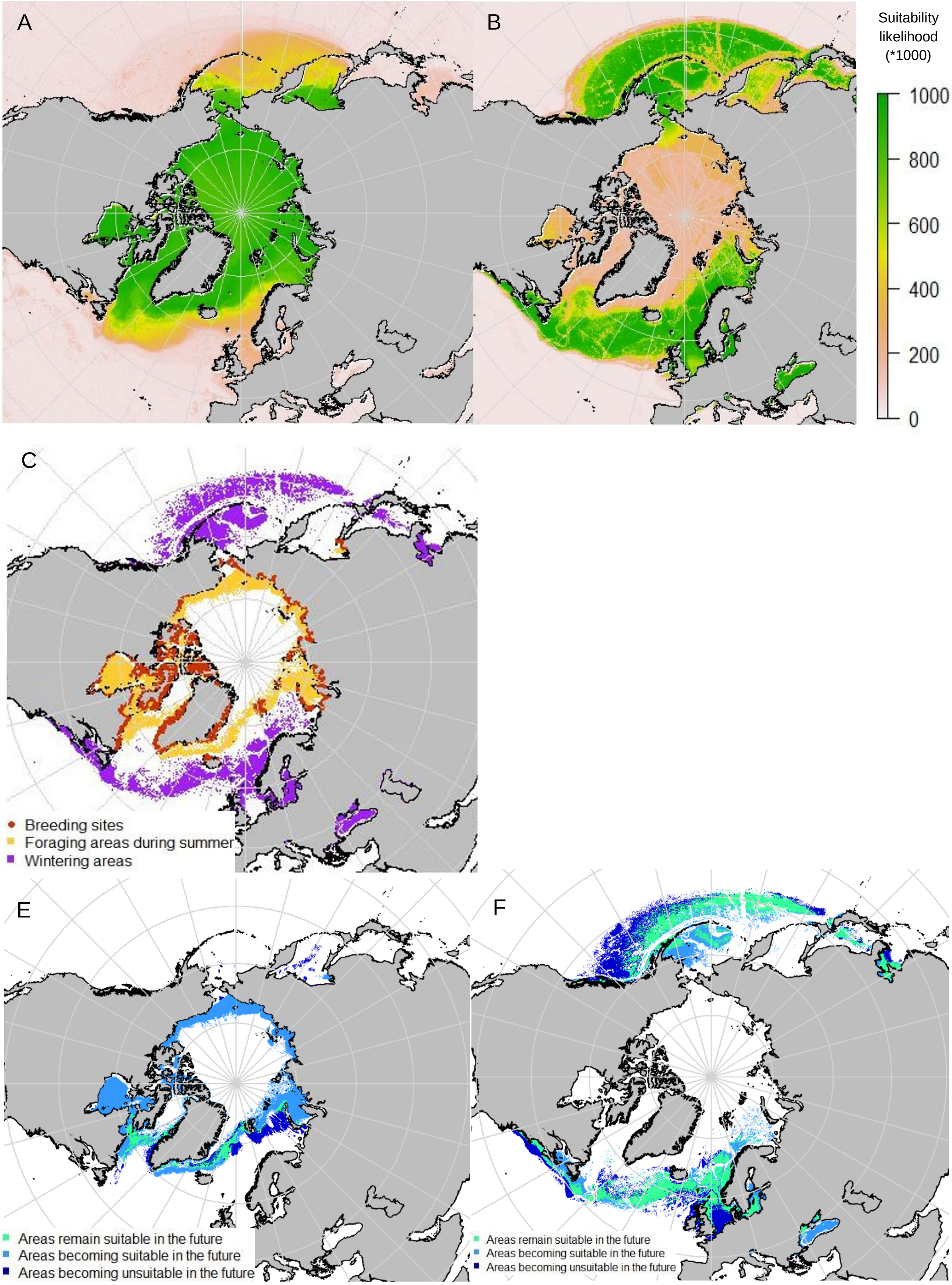

Breeding sites are those obtained with the HadGEM models (figure D is missing).

Supplemental materials VI: Coefficient of variation between climatic models for each kind of habitats

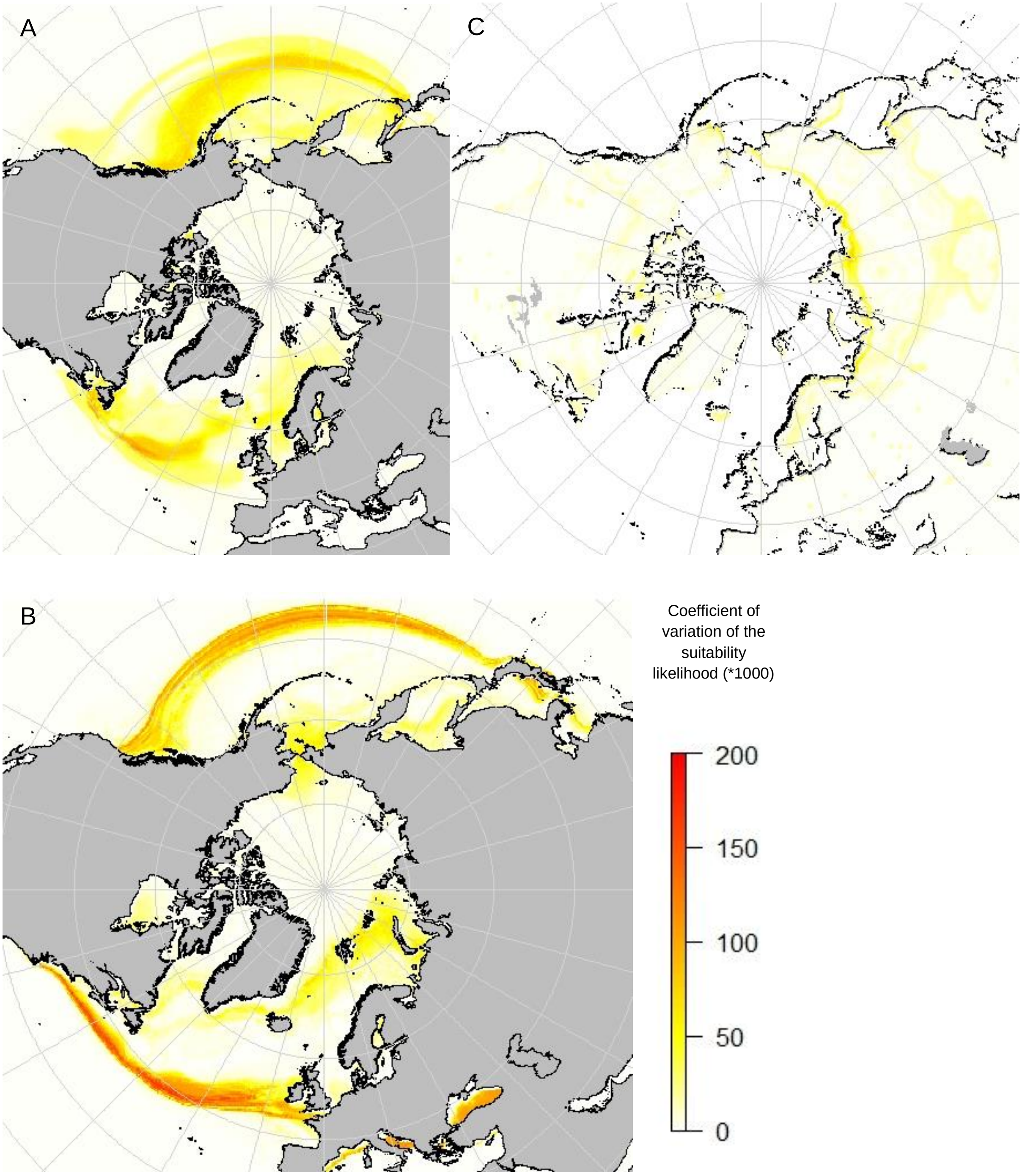

Coefficient of variation of suitability probability (*1000) for the four climatic models during summer (figure A), winter (figure B) and breeding (figure C) periods. For breeding, only HadGEM2-CC and HadGEM2-ES outputs are considered.

Supplemental materials VII: Potential suitable (suitability likelihood > 0.9) little auk breeding habitats involved in the residency strategy, currently (2000-2017) and in the future (2050-2059, RCP 8.5 scenario) according to the four climatic models.

Current projection

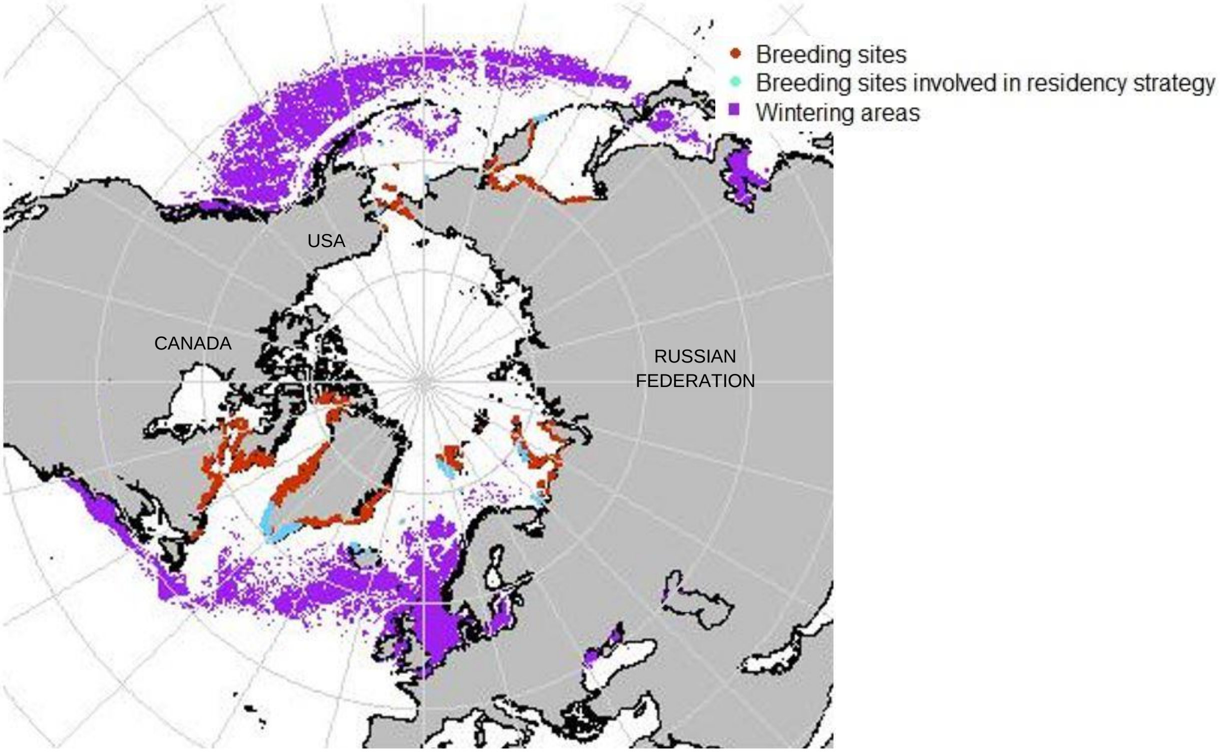

Future projection with the HadGEM2-CC climatic model

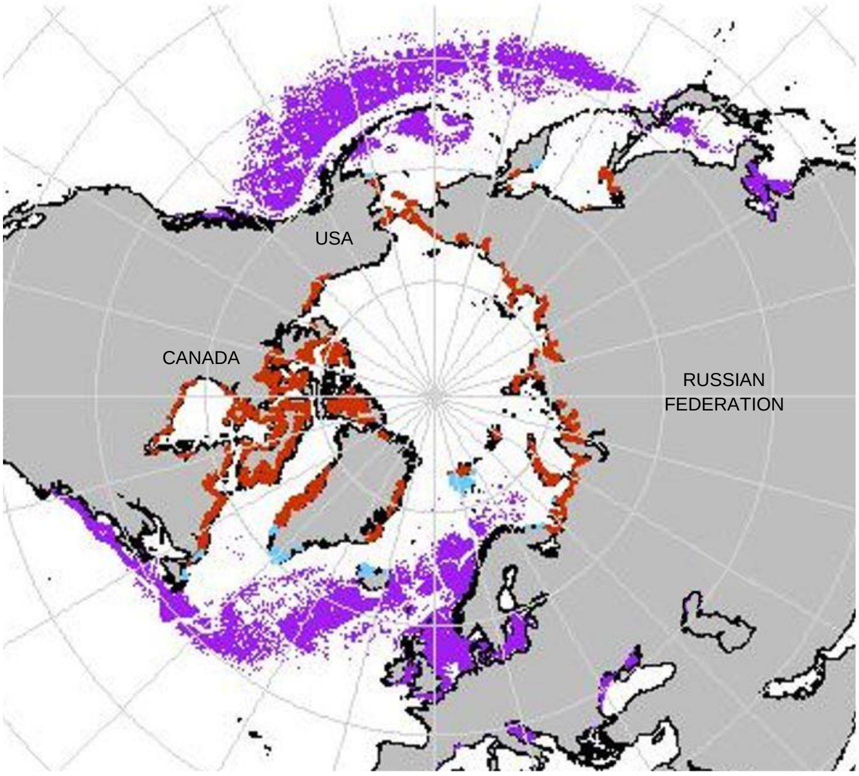

Future projection with the HadGEM2-ES climatic model

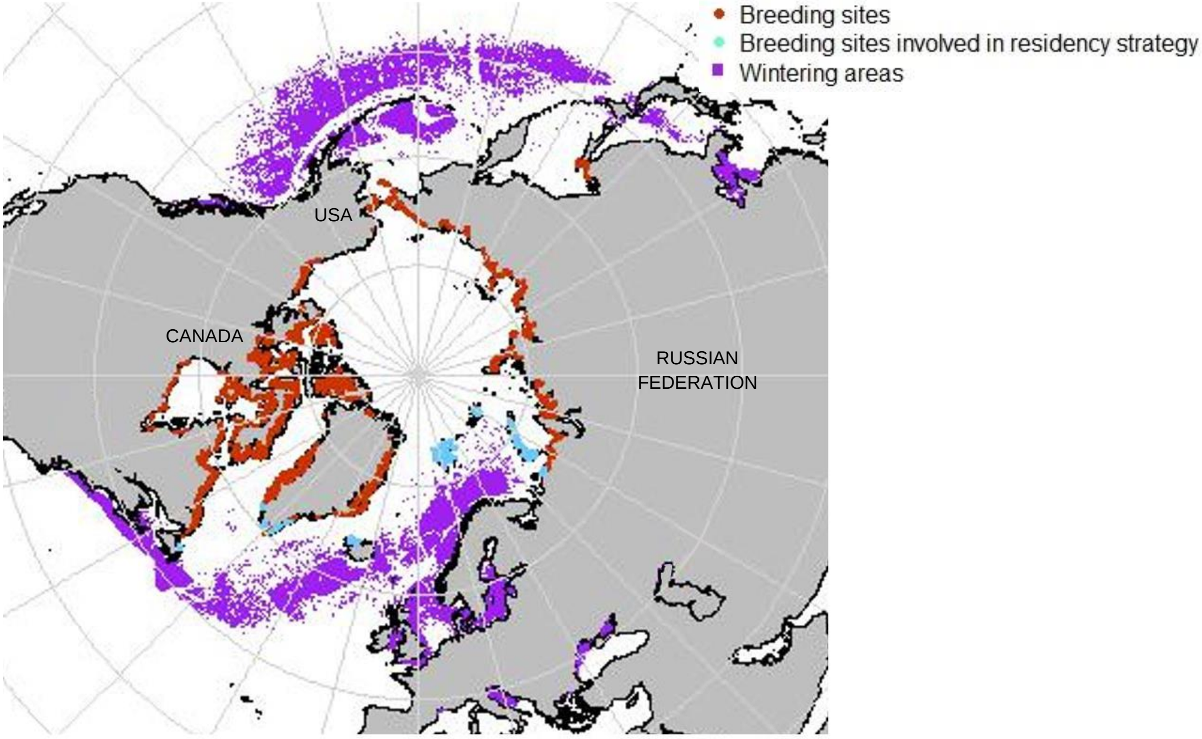

Future projection with the ACCESS 1.0 climatic model

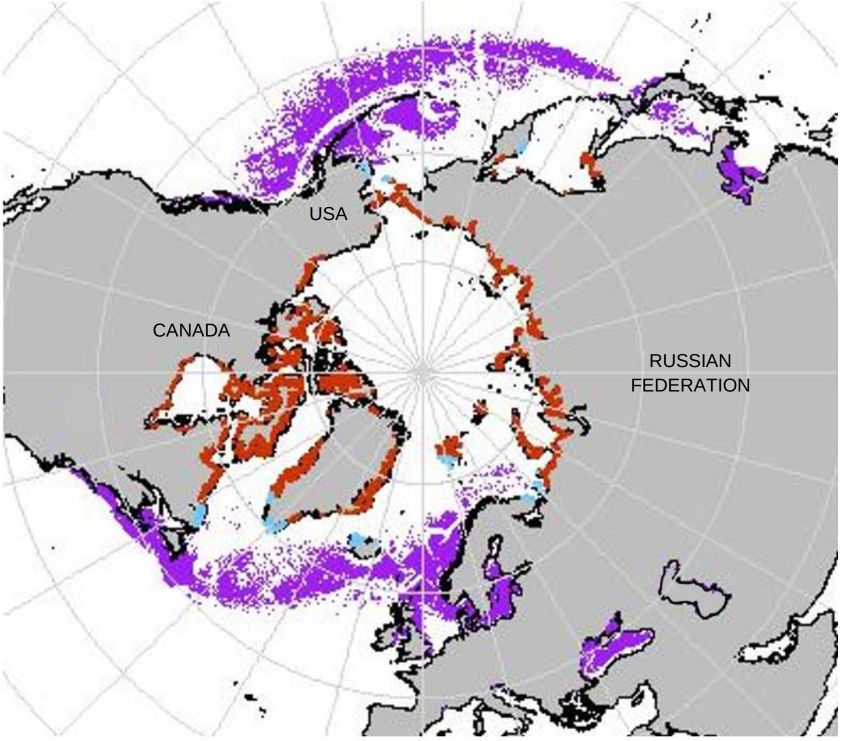

Future projection with the ACCESS 1.3 climatic model

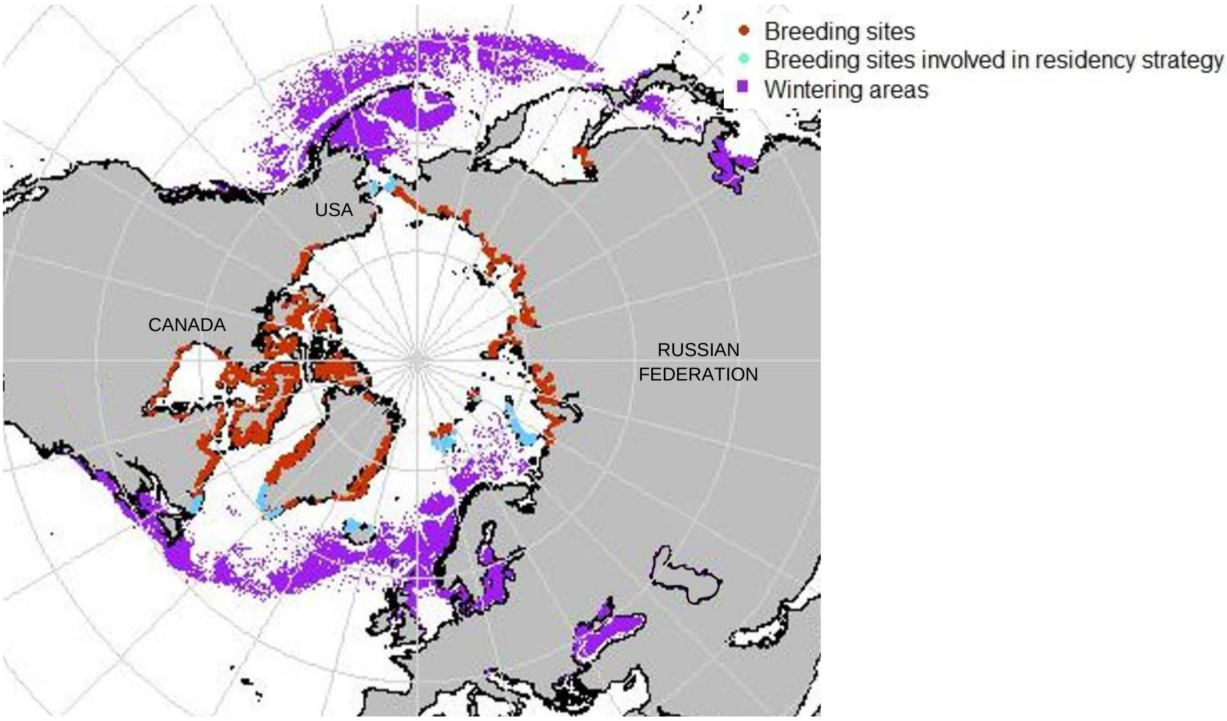

Supplement: Supplementary file 1 — Supplementary materials [file 41598_2019_54228_MOESM1_ESM.pdf]
